# Urinary metabolic variation analysis during pregnancy and application in Gestational Diabetes Mellitus and spontaneous abortion biomarker discovery

**DOI:** 10.1038/s41598-019-39259-2

**Published:** 2019-02-22

**Authors:** Xiaoyan Liu, Xiangqing Wang, Haidan Sun, Zhengguang Guo, Xiang Liu, Tao Yuan, Yong Fu, Xiaoyue Tang, Jing Li, Wei Sun, Weigang Zhao

**Affiliations:** 1 Institute of Basic Medical Sciences, Chinese Academy of Medical Sciences, School of Basic Medicine, Peking Union Medical College, Beijing, China; 2 Department of Endocrinology, Key Laboratory of Endocrinology of Ministry of Health, Peking Union Medical College Hospital, Chinese Academy of Medical Science and Peking Union Medical College, Beijing, China; 3 Department of Endocrinology and Metabolism, Peking University People’s Hospital, Peking University Diabetes Center, Beijing, China

**Keywords:** Metabolomics, Liquid chromatography

## Abstract

Pregnancy is associated with the onset of many adaptation processes that are likely to change over the course of gestation. Understanding normal metabolites’ variation with pregnancy progression is crucial for gaining insights of the key nutrients for normal fetal growth, and for comparative research of pregnancy-related complications. This work presents liquid chromatography-mass spectrum-based urine metabolomics study of 50 health pregnant women at three time points during pregnancy. The influence of maternal physiological factors, including age, BMI, parity and gravity to urine metabolome was explored. Additionally, urine metabolomics was applied for early prediction of two pregnancy complications, gestational diabetes mellitus and spontaneous abortion. Our results suggested that during normal pregnancy progression, pathways of steroid hormone biosynthesis and tyrosine metabolism were significantly regulated. BMI is a factor that should be considered during cross-section analysis. Application analysis discovered potential biomarkers for GDM in the first trimester with AUC of 0.89, and potential biomarkers for SA in the first trimester with AUC of 0.90. In conclusion, our study indicated that urine metabolome could reflect variations during pregnancy progression, and has potential value for pregnancy complications early prediction. The clinical trial number for this study is NCT03246295.

## Introduction

Pregnancy is associated with the onset of many adaptation processes that are likely to change over the course of gestation for adaptation to the fetus. The glucose, steroids, amino acids and lipids are all utilized by the fetoplacental unit, and thus maternal metabolism must adapt to ensure the fetal demands^1^. Glucose is an essential nutrient for fetus development, and pregnant women become increasingly insulin resistant, particularly from the second trimester onwards^2^. In addition, maternal lipid concentrations dramatically increase and circulating amino acid concentrations are also suggested to be altered largely in response to increased protein synthesis for placental and fetal growth during the third trimester^3,4^. Most of the metabolism changes is normal physiological responses. But in several pregnant women the changes may be off –track and become harmful, for example, abortion, gestational diabetes, and preterm birth. Understanding normal metabolites’ variation with pregnancy progression is crucial for gaining insights of the key nutrients for normal fetal growth, and for comparative research of pregnancy-related complications with abnormal metabolism.

Metabolome of biofluids, urine, serum and amniotic fluid should reflect biochemical dynamics during pregnancy progression. A few researchers have been investigating metabolite variations in pregnancy women using various approaches, including NMR and LC-MS. In 2014, Luan, Hemi *et al*., performed a LC-MS plasma metabolome based on 180 healthy pregnant women, six time points spanning all three trimesters. Biopterin metabolism, phospholipid metabolism, amino acid derivatives, and fatty acid oxidation were found to be altered with pregnancy progression. This study provided sufficient coverage to model the progression of normal pregnancy, but individual differences could affect the results due to different subjects in each trimester^5^. Later in 2015, Lindsay, K. L. *et al*., further performed a targeted plasma metabolomics to detect variations of amino acids and non-esterified fatty acids during pregnancy based on 160 pregnancy women followed until the end of their pregnancy. Plasma concentration of several essential and non-essential amino acids and long-chain polyunsaturated fatty acids significantly decreased across pregnancy^6^. A larger scale serum metabolomics study during pregnancy was performed in 2016 based on 322 healthy pregnancy and 3938 non-pregnant women^7^. Both cross-sectional and longitudinal comparisons were explored and 124 biomarkers, including 87 metabolites and 37 cytokines were quantified by a high-throughput serum NMR metabolomics platform. Substantial metabolic and inflammatory (lipoprotein subclasses and lipids) changes were occurred in the mothers. To date, very few studies have approached mapping of the changes in maternal urinary metabolomics. In 2015, seventy-two individual urine samples were examined spanning 9–23 weeks of gestation without follow-up using HILIC-MS platform^8^. Only 383 ions were identified as metabolomic components of pregnancy urine duo to the limitation of HILIC analysis range, and only a few differential metabolites, 5-phosphonooxy-L-lysine, β-guanidinopropionic acid, trihexosylceramide (d18:1/12:0) and 10-HDoHE were identified. Comprehensively longitudinal characterization of the maternal urine metabolomics is needed.

Maternal physiological variables, including age, body mass index (BMI), parity and gravity could contribute to metabolism variations during pregnancy. Therefore, perturbations in these elements must be considered during the design of experiments. It have been reported that higher maternal BMI are associated with broad-based changes in maternal serum metabolites, with lipids and lipid-related metabolites accounting for the association of maternal BMI with newborn size at birth^9^. Also association of serum lipids, amino acids and their metabolites, with maternal BMI and glucose levels was found in previous targeted serum metabolomics^10^. Besides BMI, the influence of other factors, including age, parity and gravity to maternal urine metabolome was still unknown. Previous study has suggested that age could contribute to urine metabolites variation in health subjects^11^. Whether this is occurred in pregnant women or not need to be further explored, since age range of the pregnant woman is small.

In present work, we collected 150 urine samples from 50 health pregnant women at three time points spanning all three trimesters, to significantly explore metabolome variations with pregnancy progression. The influence of maternal age, BMI, parity and gravidity on urine metabolome was explored. Our results showed that urine metabolomics could reflect metabolism variations during pregnancy. Application of urine metabolomics for pregnant complications research was further performed taking GDM and SA as models. These results suggested that urine metabolomics could reflect metabolism variations of pregnant complications in the first trimester as early as 8 week’s gestation. This study will provide the basis for time-dependent metabolic trajectory of pregnant complications.

## Results

**Figure 1:**
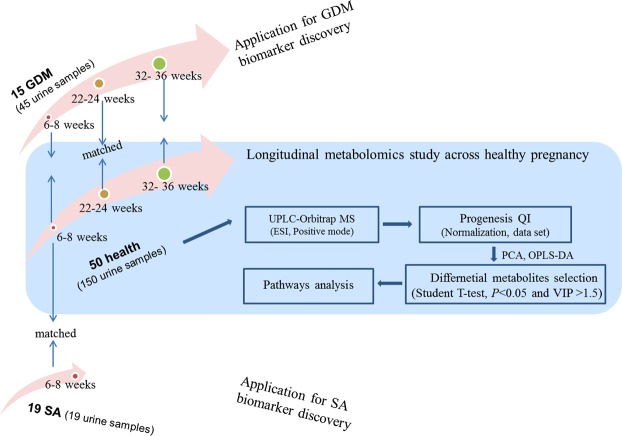
Study design.

The design of this study was shown in Fig. 1. A total of 50 health pregnant women, 15 GDM and 19 SA were finally enrolled in our study. 150 and 45 urine samples from health and GDM subjects were collected at three time points spanning the three trimesters. And 19 urine samples from 19 SA were collected at 6–8 weeks of pregnancy. Longitudinal study of metabolome variation with pregnancy progression in health pregnant women were explored. Further, urine biomarkers of GDM and SA were explored based on cross-sectional analysis of 15 GDM, 19 SA and their corresponding BMI-matched health controls (Fig. 1).

### Longitudinal metabolomics study of healthy controls with pregnancy progression

LC-MS analysis was applied for urinary metabolomics fingerprinting of the 214 samples from 84 participates (50 health, 15 GDM and 15 SA) during pregnancy. Metabolomics data quality was controlled using QC samples. The results showed that our method had essential repeatability and stability throughout the analytical run (Supplemental Materials 1 and Supplementary Fig. S1).

**Figure 2:**
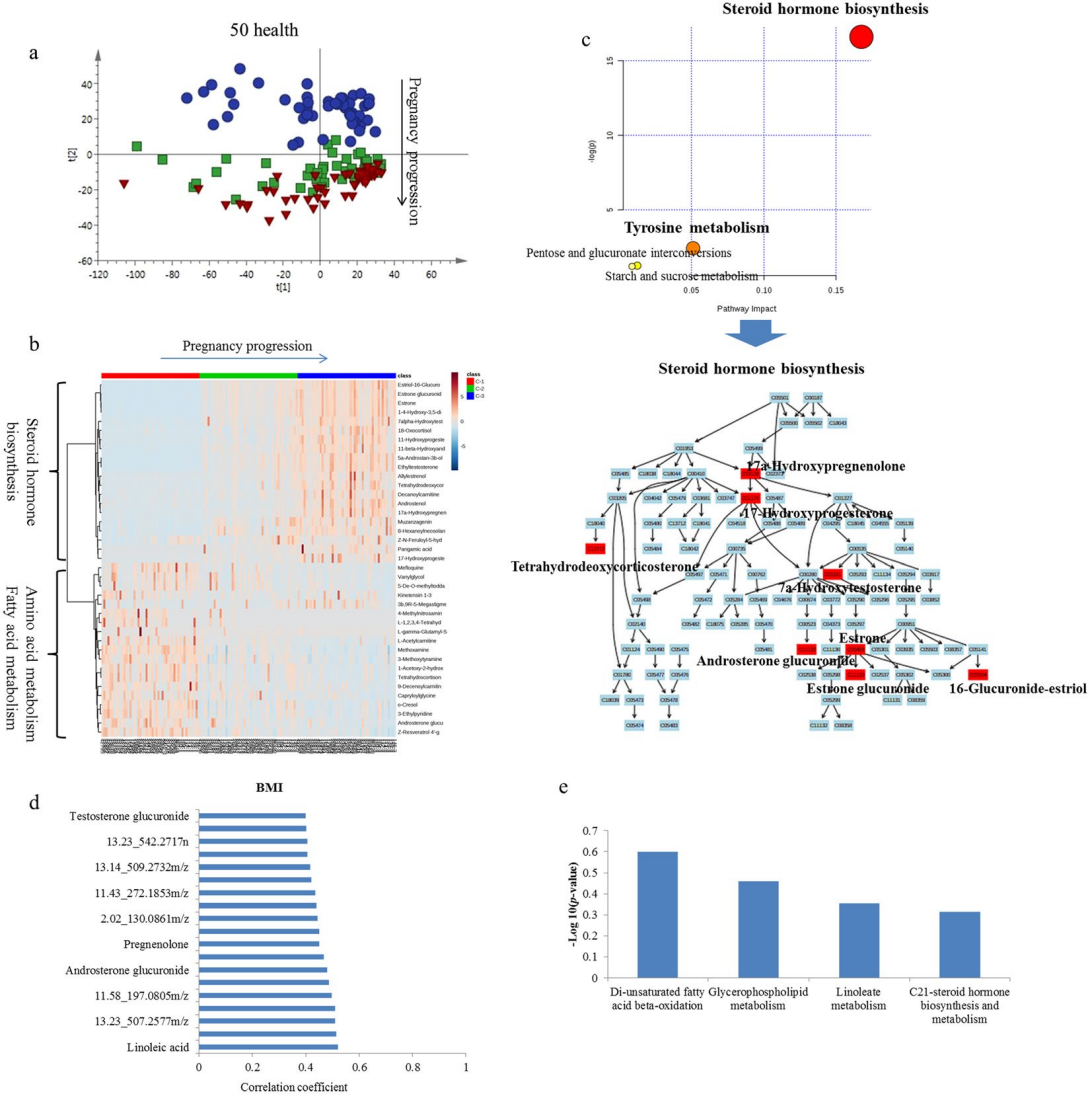
Analysis of metabolic profiling variation of health pregnancy progression. (**a**) Score plot of PCA of urinary metabolic profiling in health controls. (**b**) Heatmap of relative intensity of differential metabolites with pregnancy progression. (**c**) Pathway analysis of differential metabolites with health pregnancy progression. (**d**) Correlation coefficient of BMI and urine metabolites. The absolute value above 0.4 was referred to have medium correlation. (**e**) Enriched pathway of metabolites correlated with BMI.

Longitudinal metabolomics analysis of 50 healthy controls at different stages of gestation (one, second and third trimester) was performed to explore the metabolism change during a healthy pregnancy progression. A PCA score plot is shown in Fig. 2a. In the PCA score plot, the three gestation stages of health controls were clustered into three bands along with the PC2 axis. The first trimester differed apparently from the second and third trimester, while profiling of the second trimester overlaid mostly with that of the third trimester. These results indicated that variations of metabolic status during the second trimester and third trimester in healthy was smaller than that occurring during the first trimester and second trimester.

As this longitudinal follow up study provided significantly differential metabolic states during pregnancy progression in healthy subjects, we explored the metabolites that contributed most to normal pregnancy progression during pregnancy. The data sets were further analyzed by OPLS-DA (Supplementary Fig. S2). There was a tendency for group separation between controls both in the 1st and 2nd trimester and 2nd trimester and 3rd of trimester of gestation. Integrated SUS-plots obtained from the OPLS-DA models (first trimester vs. second trimester and second trimester vs. third trimester) were used to evaluate the correlation of metabolites with pregnancy progression in healthy subjects (Supplementary Fig. S2). One hundred permutations were used to validate OPLS-DA models, which showed no over fitting. These results showed that, along with pregnancy progression, the metabolic status showed significant differences in healthy subjects.

Using variable correlation coefficients obtained from SUS plots, combined with the ANOVA test significance (*p* < 0.05), a total of 39 metabolites were selected as the differential metabolites during pregnancy in healthy control (Supplementary Table S1). These metabolites play an important role during healthy pregnancy progression. There were two clusters: one cluster increased with pregnancy progression, such as steroid hormones and their metabolites, and another cluster decreased with pregnancy progression, such as tyrosine metabolites, fatty acid metabolites (Fig. 2b). MetPA pathway analysis showed that steroid hormone biosynthesis was upregulated in healthy controls during pregnancy with a high impact. Tyrosine metabolism was down-regulated with less impact (Fig. 2c). The steroid hormone biosynthesis pathway was mapped with MetPA. The pathway is a simplified KEGG pathway map with emphasis on chemical compounds. The identified metabolites were highlighted. Most metabolites of steroid hormone biosynthesis were upregulated in the urinary metabolome in healthy controls with pregnancy. These included 17a-hydroxypregnenolon, 17-hydroxyprogesterone, tetrahydrodeoxycorticosterone, 7a-hydroxytestosterone, estrone, estroneglucuronide and 16-glucuronide-estriol.

### Correlation of maternal physiological variables with urine metabolome

Previous study suggested correlation of physiological variables (age, sex, BMI) with urine metabolomics in health subjects^11,12^. Herein, a correlation analysis between health maternal urine metabolome and physiological variables was performed. The results indicated a medium correlation between BMI and several urine metabolites (Fig. 2d and Supplementary Fig. S3), indicated a potential role of maternal fats on urine metabolome during pregnancy. Features with medium correlation coefficient were further analyzed using “mummichog” algorithm to investigate potential associated pathways. Enrichement analysis showed that variations of glycerophospholipid metabolism, androgen and estrogen biosynthesis and metabolism and unsaturated fatty acid beta-oxidation were correlated with maternal BMI (Fig. 2e). For age, parity and gravidity, no significant correlation with urine metabolome was found in present cohort, possibly due to the narrow distribution of the participates.

### Cross-sectional study of GDM and healthy controls for biomarker discovery

To discover metabolite markers for GDM, cross-sectional comparisons were carried out on metabolic profiling of 15 GDM patients and 15 BMI- matched health subjects at the three time points of gestation, respectively. PCA analysis showed that metabolic profiling in the first trimester between the controls and GDM could be discriminated obviously with an AUC of 0.804 for controls and 0.765 for the GDM group (Supplementary Figs S4a and S5a). For metabolic profiling discrimination in the second trimester, the AUC was 0.907 for controls and 0.912 for the GDM group, which showed better discrimination than that in the first trimester (Supplementary Figs S4b and S5b). These results indicated more obvious metabolome changes occurred in the second trimester. And in the third trimester, metabolic profiling discrimination between GDM and controls was not significant, which may be caused by dietary or medical interventions (AUC-control = 0.527; AUC-GDM = 0.813, Supplementary Figs S4c and S5c). These results indicated that urine metabolome could reflect GDM progression during pregnancy. Urine metabolites could predict GDM at the first trimester. And clinical interventions could recover GDM metabolism to some extent.

**Figure 3:**
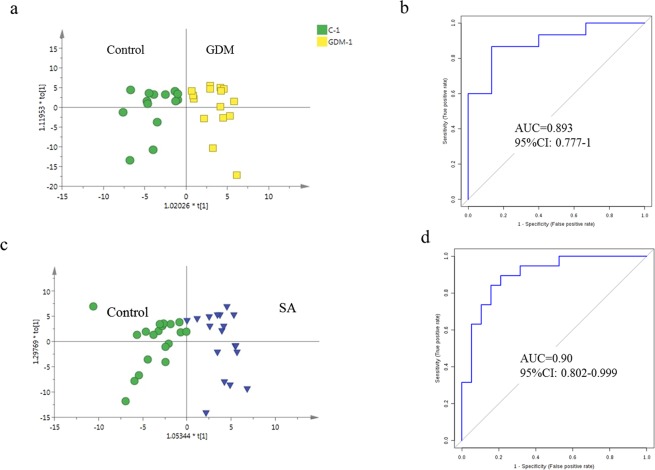
Early prediction of GDM and SA. (**a**) OPLS-DA score plot of urine metabolic of GDM and the health at first trimester. (**b**) ROC plot with 10-fold cross-Validation based on Logistic Regression Model based on metabolites L-phenylalanyl-L-proline, Hydroxylauroylcarnitine and levoglucosan. (**c**) OPLS-DA score plot of urine metabolic of SA and the health at first trimester. (**d**) ROC plot with 10-fold cross-validation based on logistic regression model of indolylacryloylglycine and L-histidine.

To explore potential biomarkers for GDM predictions at the first trimester so that early prevention could be performed to reduce damage to the fetus, differential variable selection between GDM and the healthy participants at the first trimester (approximately 8 week’s gestation) was performed. Score plots of the OPLS-DA model showed clear discrimination between controls and the GDM group (Fig. 3a). Metabolites with VIP values above 1.5 were selected as potential biomarkers. Overall, 7 potential early biomarkers were identified with an AUC above 0.7 (Supplementary Table S2). Considering that more than one metabolite usually has a better diagnosis value than only one metabolite, a multivariate ROC curve based exploratory analysis was carried out (http://www.metaboanalyst.ca/faces/Secure/upload/RocUploadView.xhtml). A metabolite panel, comprised of L-phenylalanyl-L-proline, 2-hydroxylauroylcarnitine and levoglucosan was used to construct the most robust predictive model for early GDM prediction using a logistic regression algorithm. On the training set, this model classified GDM and controls with the AUC of 0.96, and the 10-fold cross-validation AUC value was 0.89 (Fig. 3b). The sensitivity and the specificity were above 0.85, which showed the robustness of the predictive model (Table 1).

**Table 1:** Prediction ability of early clinical differential data and metabolite panel for GDM and SA.

Parameter		AUC	Sensitivity	Specificity
**GDM early prediction (~8 week’s gestation)**				
Urine Metabolites panel (L-phenylalanyl-L-proline + Hydroxylauroylcarnitine + levoglucosan)	Training/Discovery	0.96 (0.934–0.980)	93.3%	89.6%
	10-fold Cross-Validation	0.89 (0.777–1.000)	86.7%	86.7%
**SA early prediction (~8 week’s gestation)**				
Urine Metabolites panel (Indolylacryloylglycine + L-histidine)	Training/Discovery	0.94 (0.914~0.960)	84.2%	89.5%
	10-fold Cross-Validation	0.90 (0.802~0.999)	84.2%	84.2%

### Cross-sectional study of Spontaneous abortion and healthy controls for biomarker discovery

SA is commonly occurred during the first trimester of pregnancy. And it is difficult to discover in a timely fashion. In present study, PCA analysis showed that metabolic profiling between 19 SA women and 19 BMI- matched health pregnant women was significantly different (Supplementary Fig. S6). Further, potential biomarkers for SA prediction were selected based on OPLS-DA model. Score plot showed more clear distinction between SA and the control (Fig. 3c). Permutation validation suggested no over fitting of OPLS-DA model with R2 of 0.762, Q2 of 0.432 and the Q2-intercept of – 0.28. Based on VIP value (>1.5), overall 13 metabolites were identified as significant contributors for group discrimination (Supplementary Table S3).

Classical univariate ROC curve analyses were performed using the 13 selected marker candidates. The results showed that 11 metabolites could discriminate SA well with AUC values above 0.7 (Supplementary Table S4). To achieve high prediction accuracy, a metabolite panel consisted of indolylacryloylglycine and L-histidine was selected to construct logistic regression model. The AUC value was 0.94 for the training set. To limit overfitting, the training set was subjected to a 10-fold cross validation to evaluate the stability and generalization of the prediction model. And the cross validation reported AUC of 0.90 (Fig. 3d), with the sensitivity of 0.84 and specificity of 0.84 (Table 1). These results suggested the potential value of significant metabolites for SA discrimination.

## Discussion

In this study, we conducted a comprehensive characterization of maternal urine metabolome from a cohort of 50 pregnant women. Longitudinal metabolome variation along with pregnancy was investigated. And a biomarker exploration for SA and GDM prediction as early as about 8 week’s gestation was performed. This study would provide the basic urine metabolome characteristics of pregnancy for further pregnant complications research. And we hypothesized that the urine metabolite profile would help to diagnose GDM and SA as early as about 8 week’s gestation.

Pregnancy is associated with the onset of many adaptation processes that are likely to change over the course of gestation, including multiple steroid hormones and amino acid metabolic cycle^13^. MS-based metabolomics is a high-through technique for studying complex biological samples (e.g., blood and urine) to describe dynamic changes in phenotype and system homeostasis. Our longitudinal study suggested that metabolites involved in steroid hormone biosynthesis pathway were up-regulated with pregnancy progression in both health and GDM women. These related metabolites of estrogen, progestin, androgen and cortex hormone were all increased with pregnancy progression, except two terminal metabolites tetrahydrodeoxycorticosterone and androsterone glucuronide, which showed decreasing trend, probably resulting from the accumulation of the Intermediate metabolites. Early in previous research, it has been detected that steroid hormones, including estradiol, hydroxyprogesterone cortisol and androstenedione in serum and urine were increasing with pregnancy progression using target LC-MS method^14^. In urine, metabolites of steroid hormones, such as 17-hydroxypregnenolone and deoxycorticosterone, were found to be up-regulated during pregnancy^15^. These researches showed consistent results with ours that during normal pregnancy, function of steroid hormones biosynthesis was more activated. Steroid hormones play important roles for maintenance of placental function and regulation of pregnancy process^16,17^. Disturbance of these hormones will influence metabolism and immune functions of pregnant women, and also related to the occurrence of some gestational complications.

Except for steroid hormones, amino acids were found to be another changed metabolites with health pregnancy progression, likely reflects placental uptake and tissue biosynthesis^6^. Previous researches indicated amino acid reduction with the progression of pregnancy in amniotic fluid, which was associated with necessary for protein synthesis during fetal rapid growth^18^. Our longitudinal analysis showed down regulation of tyrosine metabolism. It showed the reduction of tyrosine metabolism during pregnancy may be derived from protein breakdown and phenylalanine hydroxylation^19^. Also, tyrosine metabolism disorder may be a risk factor of insulin resistance and gestational diabetes^20^.

Since maternal physiological factors, including age, BMI, parity and gravidity could contribute to urine metabolome variations, we explored the correlation of these factors with urine metabolites. The correlation coefficient above 0.4 was referred to be medium correlated. Our results suggested several metabolites correlated with age, BMI, parity and gravidity, respectively. However, only metabolites correlated with BMI could enriched to several metabolism pathways using “mummichog” algorithm. We suggested that these metabolites would have biological value, and should be considered during cross-analysis design. The main differential pathways affected by BMI is glycerophospholipid metabolism, androgen and estrogen biosynthesis and metabolism and unsaturated fatty acid beta-oxidation, possibly duo to the shared genetic or unhealthy eating and feeding fats diets^21^ rather than to intrauterine mechanisms^22^. In addition, BMI is one of the important risk factors of pregnancy complications, such as GDM^23^. It showed women with larger BMI in the pre-pregnancy or early gestation have higher risk of GDM occurrence^24^. Age is an important factor affection urine metabolome^11^. However, present study showed a small correlation of age and maternal urine metabolites. We suggested this is resulted from the small range of age distribution. Most (88%) of the women are at the gestational age from 26 to 36. In conclusion, BMI is an important factor that should be considered during cross-section experiment design, and age could be negligible if the enrolled subjects are in normally gestational age. Parity and gravidity contribute less to urine metabolome variation.

GDM was clinically diagnosed during the second trimester with OGTT test. GDM diagnosis as early as possible of pregnancy would be valuable for therapeutical intervention and could induce the possible damage to fetal growth and maternal health. Additionally, in the early pregnancy stage, the influence from embryo is so less that the metabolism disorders in pregnant women will be exposed independently.

Urine metabolomics, as a practical technique to identify potential biomarkers of disease, has been used for a diagnostic purpose or risk stratification for GDM. In 2012, a large cohort study, including 823 healthy, pregnant women was performed using NMR-based urine metabolomics. This study could differentiate between time points during and after pregnancy and thus track its development, but that it could not identify reliable biomarkers for gestational diabetes mellitus (GDM) in a large, multiethnic population possible due to low sensitivity of NMR and the large diurnal and dietary variation^25^. In 2017, Kai P. La performed a comprehensive longitudinal metabolomics study on 27 subjects with GDM using LC-MS based urine and plasma metabolomics^26,27^. It showed metabolites involved in tryptophan and purine metabolism could predict GDM with AUC of 0.7~0.8 at 10–14 weeks of gestation. In this study, factors of age and BMI were not matched during cross-section biomarker analysis, and their interferences to the conclusions could not be excluded, thus further validation was necessary. In present study, urine samples in the first trimester were collected at the time point of 6~8 week gestation, which was the earliest compared to previous study design^27,28^. This would be more valuable for early GDM diagnosis.

A metabolite panel consisted of L-phenylalanyl-L-proline, 2-hydroxylauroylcarnitine and levoglucosan was used to construct the most robust predictive model for early GDM prediction. The AUC was 0.96, the sensitivity was 93.3% and the specificity was 89.6%. The positive likelihood ratio was 14.1 and the negative likelihood ratio was 0.08. The accuracy is significantly higher than the reported metabolites biomarkers^29,30^. 2-hydroxylauroylcarnitine is an acylcarnitine metabolites that mianly involved in fatty acid oxidation. It suggested that serum acylcarnitines was associated with worse glucose intolerance and the long-chain fatty acid oxidation pathway was disturbed in the natural course of type 2 diabetes^31^. In GDM patients, serum acylcarnitines differences were found to be already existent in the first trimester of the pregnancy^32^, indicated the potential value for GDM prediction. Moreover, urine sample collection time in our study was as early as 8 week’s gestation, which is the earliest collection time to our knowledge. Therefore, the metabolic status is closer to pre-pregnancy and would be more valuable for early GDM diagnosis. Correlation analysis showed that level of early potential biomarker, 6-Hydroxy-5-methoxyindole glucuronide was positively correlated with OGTT (r = 0.619, p = 0.018). 6-Hydroxy-5-methoxyindole glucuronide is a natural human metabolite of 6-hydroxy-5-methoxyindole generated in the liver by UDP glucuonyltransferase. Glucuronidation is used to assist in the kidney excretion of toxic substances, drugs or other substances. It showed that activity of UDP glucuonyltransferase, catalyzing glucuronidation process was increased in diabetes mellitus mice model^33^. GDM is considered as the pre-diabetes stage, which may has similar mechanism.

SA is commonly occurred in the first trimester of gestation. Chromosome abnormality, endocrine disorder, infection, self- immune abnormities, and the environment factor all could induce termination of embryonic development. In clinic, the common monitoring indicators include progesterone, estradiol, human chorionic gonadotropin (HCG), and thyroid related antibodies, but the accuracy is not satisfied. To our knowledge, no studies explored urine metabolomics characteristics in SA women currently, let alone urine biomarker discovery. In present research, potential urinary biomarkers for early SA prediction were discovered using comparable analysis of metabolome of SA women and BMI - matched normal pregnant women. This is the first urine metabolomics study of SA patients in the early pregnancy stage. It found that metabolites involved in antioxidant stress were down-regulated in SA women compared with that in normal pregnant women, indicating the decreased toxins scavenging ability in SA women. A metabolite panel consisted of indolylacryloylglycine and L-histidine was used to construct the most robust predictive model for early SA prediction with AUC of 0.94. Indolylacryloylglycine is a metabolite of tryptophan metabolism. It is likely a product of gut metabolism. Previously study suggested that tryptophan metabolism was an important potential target pathways for missed abortion^34^. Change of urinary indolylacryloylglycine level could be the indicator of target tryptophan metabolism disturbance in SA patients. L-histidine is an essential amino acid playing multiple biological functions. L-histidine is attributed to the capacity of the imidazole ring to scavenge reactive oxygen species (ROS) generated by cells during acute inflammatory response. Low plasma concentrations of histidine are found to be associated with protein-energy wasting, inflammation and oxidative stress in obese women^35^. Decreased level of urine histidine in SA patients may indicated possible risk factors, including inflammation, oxidation stress that contribute to SA occurrence. Owning to complicated mechanism for SA occurrence and unclear causes of SA participates in this cohort, the potential value of these metabolite biomarkers need to be further validated.

In conclusion, we performed a cohort study of maternal urine metabolome in 50 health pregnant women. Further, a pilot application study was performed for potential biomarker discovery of two pregnant complications diseases, GDM and SA. The results showed that urine metabolomics could reflect pregnancy progression and could be used for pregnancy complication biomarker discovery. However, the sample size in present study is relatively small, and further large-scale and multi-center study need to be performed based on a larger prospective cohort in the future, which would be our future project. This study will provide a comprehensive urinary metabolomics basis for pregnancy complication researches.

## Materials and Methods

### Materials

Acetonitrile, formic acid and water (LC-MS grade) were obtained from Fisher Scientific. An LTQ-orbitrapvelos mass spectrometer from ThermoFisher (Framingham, MA, USA) and an ACQUITY H-class UPLC system from Waters (Milford, MA, USA) were used.

### Study design

The International Ethics Code of this study is ZS-976. This study was approved by the Ethics committee of the Beijing union hospital and conducted under the guidance of Major New Drugs Innovation and Development Program. Pregnant women who attended their first prenatal visit in the obstetric department at the Peking Union Hospital, during their first 6–10 weeks of gestation from October 2015 to May 2016 were enrolled. An obstetrician informed the eligible participants about the nature of the study and invited them to participate. All human subjects provided informed consent before participating in this study. The clinical trial number is NCT03246295. Women were grouped according to the following criteria. (1) Normal groups (50 subjects): Participates without diabetes and GDM history; All the blood-glucose tests during pregnancy were in normal range. (2) GDM group (15 subjects): participates without diabetes history; a routine oral glucose tolerance test (OGTT) was performed at 24–28 weeks of gestation, and one or more items exceed the threshold value was diagnosed with GDM: fasting blood-glucose (FBG) ≥ 5.1 mmol/L, 1 hour blood-glucose ≥ 10.0 mmol/L, 2 hour blood-glucose ≥ 8.5 mmol/L. (3) SA group (19 subjects): women these stopped embryo development during the first trimester. Women diagnosed with GDM were referred to nutritionists and were given advice on dietary and lifestyle interventions for controlling their blood glucose.

### Urine Sample Preparation

Midstream urine samples were collected from the participants for specimen collection. The samples were aliquoted (1 ml), frozen immediately, and stored at −80 °C until further processing. Acetonitrile (200 μl) was added into each urine sample (200 μl), and then the mixture was vortexed for 30 s and centrifuged at 14,000 × g for 10 min. The supernatant was dried under a vacuum and then reconstituted with 200 μl of 2% acetonitrile. Urinary metabolites were further separated from larger molecules using 10 kDa molecular weight cut-off (MWCO) ultracentrifugation filters (Millipore Amicon Ultra, MA) before being transferred to the autosamplers. The quality control (QC) sample was a pooled urine sample prepared by mixing equal aliquots of all samples to be analyzed, which were therefore globally representative of the whole sample set. The QC samples were injected every ten samples throughout the analytical run to provide a set of data from which method stability and repeatability could be assessed.

### LC-MS analysis

Ultra-performance LC-MS analyses of urine samples were conducted using a Waters ACQUITY H-class LC system coupled with an LTQ-Orbitrap mass spectrometer (Thermo Fisher Scientific, Villebon-sur-Yvette, France). Urinary metabolites were separated with a 29 min gradient on a Waters HSS C18 column (3.0 × 100 mm, 1.7 μm) at a flow rate of 0.3 ml/min. Mobile phase A was 0.1% formic acid in H2O and mobile phase B was acetonitrile. The gradient was set as follows: 0–2 min, 2% solvent B; 2–5 min, 2–55% solvent B; 5–15 min, 55–100% solvent B; 15–20 min, 100% solvent B; 20–20.1 min, 100-2% solvent B; 20.1–29 min, 2% solvent B. The column temperature was set at 50 °C. Full MS acquisition was achieved with scanning from 100 to 1000 m/z at a resolution of 60 K. UPLC targeted-MS/MS analyses were acquired at a resolution of 15 K. Collision energy was optimized as 20, 40, 60 or 80 for each target with higher-energy collisional dissociation (HCD) fragmentation. The injection order of urine samples with 3 technical replicates was randomized to reduce the experimental bias.

### Data analysis

Metabolite extraction was conducted with Progenesis QI (Waters, Milford, MA, USA) software, including peak alignment, peak picking and normalization to total compounds. Further data preprocessing including missing value estimation (50% rule), log transformation and Pareto scaling were carried out using MetaboAnalyst 3.0 (http://www.metaboanalyst.ca). Variables whose CV% (coefficient of variations) were more than 30% and missed in 50% samples were removed from further statistical analysis. Pattern recognition analysis (principal component analysis, PCA; partial least squares discriminate analysis, PLS-DA and orthogonal partial least squares discriminant analysis,OPLS-DA) was carried out using SIMCA 14.0 (Umetrics, Sweden) software. Non-parametric tests (Wilcoxon rank-sum test) were used to evaluate the significance of variables between disease and control groups. And no parameter ANOVA (Fisher-s LSD, Post-hoc analysis) were used for metabolites comparison of the three pregnant stages. False discovery rate (FDR) correction was used to estimate the chance of false positives and correct for multiple hypothesis testing. The adjusted p-value (FDR) cutoff was set as 0.05. Pearson r based distance analysis was used to assess the correlation of clinical and physiological parameters with urine metabolome.

Identification of the significant metabolites based on the Progenesis QI vehicle was performed based on a published identification strategy^36^. The exact masses of monoisotopic molecular weights were used to search Progenesis QI embedded databases, including HMDB (http://www.hmdb.ca/), ChemSpider (http://www.chemspider.com), KEGG (http://www.genome.jp) and fragmentation database (http://www.lipidmaps.org). Putative annotation of the differential compounds was performed according to multiple parameters, including isotope similarity, mass error and fragmentation score. Score ranging from 0 to 60, is used to quantify the reliability of each identity. The threshold was set at 35.0. Pathway analysis was performed with MetaAnalyst 3.0, a web-based tool for visualization of metabolomics. Predictive accuracy of biomarkers was assessed using ROC curve plotted in MetaAnalyst 3.0.

## Supplementary information

Online Supplemental Materials

## Data Availability

The datasets analyzed during the current study are available from the corresponding author on reasonable request.
