# Author Correction: Urinary metabolic variation analysis during pregnancy and application in Gestational Diabetes Mellitus and spontaneous abortion biomarker discovery

**DOI:** 10.1038/s41598-019-54733-7

**Published:** 2019-11-26

**Authors:** Xiaoyan Liu, Xiangqing Wang, Haidan Sun, Zhengguang Guo, Xiang Liu, Tao Yuan, Yong Fu, Xiaoyue Tang, Jing Li, Wei Sun, Weigang Zhao

**Affiliations:** 1 Institute of Basic Medical Sciences, Chinese Academy of Medical Sciences, School of Basic Medicine, Peking Union Medical College, Beijing, China; 2 Department of Endocrinology, Key Laboratory of Endocrinology of Ministry of Health, Peking Union Medical College Hospital, Chinese Academy of Medical Science and Peking Union Medical College, Beijing, China; 3 Department of Endocrinology and Metabolism, Peking University People’s Hospital, Peking University Diabetes Center, Beijing, China

Correction to: *Scientific Reports* 10.1038/s41598-019-39259-2, published online 22 February 2019

This Article contains errors in the Discussion section,

“In 2017, Kai P. La performed a comprehensive longitudinal metabolomics study on 27 subjects with GDM using LC-MS based urine and plasma metabolomics^26,27^. It showed metabolites involved in tryptophan and purine metabolism could predict GDM with AUC of 0.7~0.8 at 10–14 weeks of gestation. In this study, factors of age and BMI were not matched during cross-section biomarker analysis, and their interferences to the conclusions could not be excluded, thus further validation was necessary. In present study, urine samples in the first trimester were collected at the time point of 6~8 week gestation, which was the earliest compared to previous study design^27,28^. This would be more valuable for early GDM diagnosis.”

should read:

“In 2017, Kai P. Law performed a comprehensive longitudinal metabolomics study on 27 subjects with GDM using LC-MS based urine and plasma metabolomics^26,27^. It showed metabolites involved in tryptophan and purine metabolism could predict GDM with AUC of 0.7~0.8 at 10–14 weeks of gestation. It was generally considered that GDM urine was an inferior biological matrix relative to plasma in revealing the physiological changes induced by GDM since the biological variation of urine samples are typically large. However, Kai P. Law and his colleagues successfully performed comprehensive longitudinal metabolomics studies not only using GDM plasma but also on GDM urine, and demonstrated the clinical value of urinary metabolomics in the investigation of GDM. We explored the question further with GDM urine using methods familiar to clinicians/clinical scientists. In present study, urine samples in the first trimester were collected at the time point of 6~8 week gestation, which was the earliest compared to previous study design^27,28^. This would be more valuable for early GDM diagnosis.”

