# Supplementary material for: Urinary metabolic variation analysis during pregnancy and application in Gestational Diabetes Mellitus and spontaneous abortion biomarker discovery
Source: Sci Rep. 2019 Feb 22;9:2605. doi: 10.1038/s41598-019-39259-2 (PMC6384939; doi:10.1038/s41598-019-39259-2)
Supplement: Supplementary file 1 — Online Supplemental Materials [file 41598_2019_39259_MOESM1_ESM.docx]

**Online Supplemental Materials**

**Urinary metabolic variation analysis during pregnancy and application in Gestational Diabetes Mellitus and spontaneous abortion biomarker discovery**

Xiaoyan Liu*^1#^*, Xiangqing Wang *^2,3#^*，Haidan Sun*^1^*， Zhengguang Guo*^1^*， Xiang Liu*^1^*，Tao Yuan*^2^*，Yong Fu *^2^*，Xiaoyue Tang*^1^*，Jing Li*^1^*, Wei Sun*^1 *^*, Weigang Zhao *^2*^*

*^1^* Institute of Basic Medical Sciences, Chinese Academy of Medical Sciences, School of Basic Medicine, Peking Union Medical College, Beijing, China

*^2^* Department of Endocrinology, Key Laboratory of Endocrinology of Ministry of Health, Peking Union Medical College Hospital, Chinese Academy of Medical Science and Peking Union Medical College, Beijing, China

*^3^* Department of Endocrinology and Metabolism, Peking University People’s Hospital , Peking University Diabetes Center, Beijing, China

***Corresponding author:** Prof. Wei Sun, E-mail: [sunwei1018@hotmail.com](mailto:sunwei1018@hotmail.com); Tel: 0086-010-69156995

Prof. Weigang, Zhao , E-mail: xiehezhaoweigang@163.com; Tel: 0086-010-69155100

*^#^* These authors contributed equally to this work

**Supplementary Figures**

**S**[**upplementary**](file:///D:\Program%20Files%20(x86)\Youdao\Dict\7.2.0.0703\resultui\dict\?keyword=supplementary) [**materials**](file:///D:\Program%20Files%20(x86)\Youdao\Dict\7.2.0.0703\resultui\dict\?keyword=materials) **1** Metabolomics data Quality control

**Fig. S1** Assessment of QC samples. a. Trend plot showing the variation of t [1] over all QC Samples. X axis numbers represented sample number , Y axis was arbitrary (3 s.d.); b. PC1 versus PC2 of test samples and QC samples. Circle, test samples; Inverted triangle, QC samples. c. Variation distributions of features among test samples (SV%) and QC samples (CV%).

**Fig. S2** OPLS-DA analysis of pregnancy progression in health control (a (Model1) and b (Model 2)) and GDM patients (d (Model 3) and e (Model 4)). c. SUS-plot based on a and b models. Variables located on the arrow regions showed linear changes with pregnancy progression, and the bigger the absolute value of X and Y, the stronger the correlation with pregnancy progression. f. SUS-plot based on d and e models.

**Fig. S3** Correlation coefficient of BMI and urine metabolites. The absolute value above 0.4 was referred to have medium correlation.

**Fig. S4** PCA analysis of metabolic profiling between the control and GDM in the three trimesters. a. Score plot of metabolic profiling in the first trimester between the control and GDM. b. Score plot of metabolic profiling in the second trimester between the control and GDM. c. Score plot of metabolic profiling in the third trimester between the control and GDM

**Fig. S5** Analysis of metabolic profiling discrimination between the control and GDM during the three trimesters. a. ROC plots based on urine metabolic profiling to quantify the discrimination degree of the first trimester between control and GDM. b. ROC plots based on urine metabolic profiling to quantify the discrimination degree of the second trimester between control and GDM. c. ROC plots based on urine metabolic profiling to quantify the discrimination degree of the third trimester between control and GDM

**Fig.S6** PCA score plot of metabolic profiling in the first trimester between the control and SA.

**Fig S7 MS2 spectra of the putatively annotated metabolites extracted by Progenesis QI**

**Supplementary Tables**

**Table S1** Differential metabolites during health pregnancy progression

**Table S2** Information of seven potential biomarkers in the first trimester of the health and GDM

**Table S3** Differential metabolites in SA

**Table S4** Information of potential biomarkers of SA prediction

**S**[**upplementary**](file:///D:\Program%20Files%20(x86)\Youdao\Dict\7.2.0.0703\resultui\dict\?keyword=supplementary) [**materials**](file:///D:\Program%20Files%20(x86)\Youdao\Dict\7.2.0.0703\resultui\dict\?keyword=materials) **1**

**Metabolomics data Quality control**

To obtain reliable and high quality data, strict steps were taken to reduce the technical variation. During UPLC-MS analysis, samples from different groups were arranged in random order in the analysis batch. The separation and detection conditions were well controlled. The quality of QC data could also be assessed by their clustering and their variability with respect to run order. The PCA first component for the QC samples versus time analyzed was < ± 2SD, which proved good repeatability and stability during analysis process (Fig S1a). The tight clustering of QC samples (Fig. S1b) further demonstrated the quality of QC data. The mean CV of features was 1.4 in all the urinary metabolome, and these features showed a mean technical CV of 0.16 (Fig. S1c). This type of results provided some assurances that the method had essential repeatability and stability throughout the analytical run and the differences among groups were proved to be more likely to reflect varied metabolites rather than technical variations.


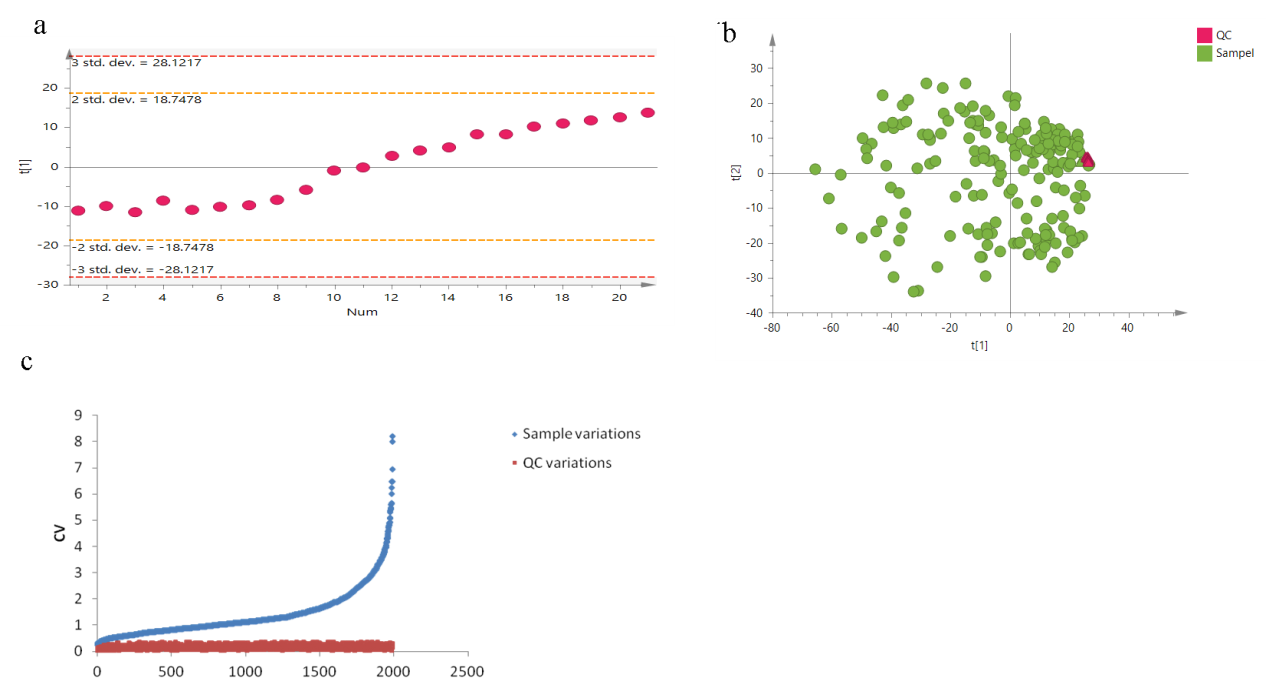


**Fig. S1.** Assessment of QC samples. a. Trend plot showing the variation of t [1] over all QC Samples. X axis numbers represented sample number , Y axis was arbitrary (3 s.d.); b. PC1 versus PC2 of test samples and QC samples. Circle, test samples; Inverted triangle, QC samples. c. Variation distributions of features among test samples (SV%) and QC samples (CV%).

**Fig. S2** OPLS-DA analysis of pregnancy progression in health control (a (Model1) and b (Model 2)). c. SUS-plot based on a and b models. Variables located on the arrow regions showed linear changes with pregnancy progression, and the bigger the absolute value of X and Y, the stronger the correlation with pregnancy progression.


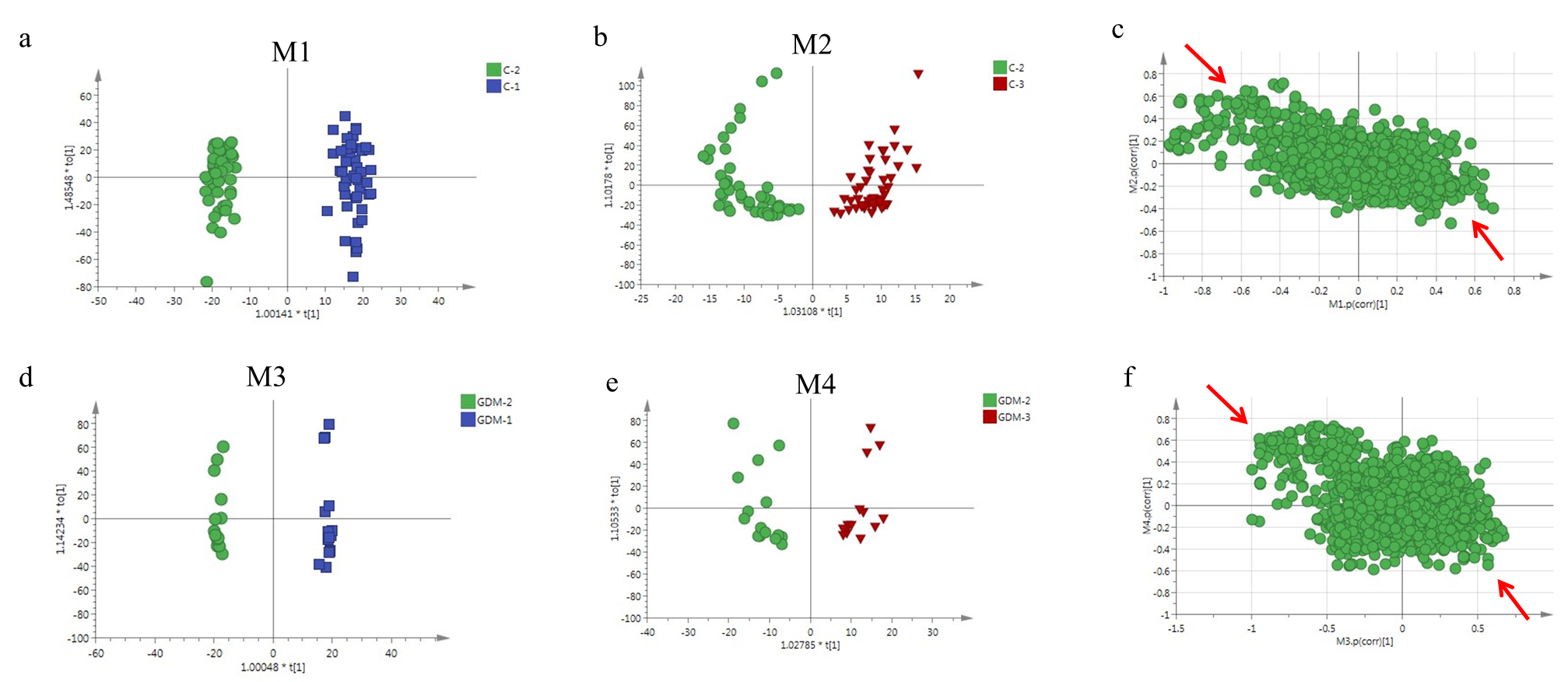


**Fig. S3** Correlation coefficient of BMI and urine metabolites. The absolute value above 0.4 was referred to have medium correlation.


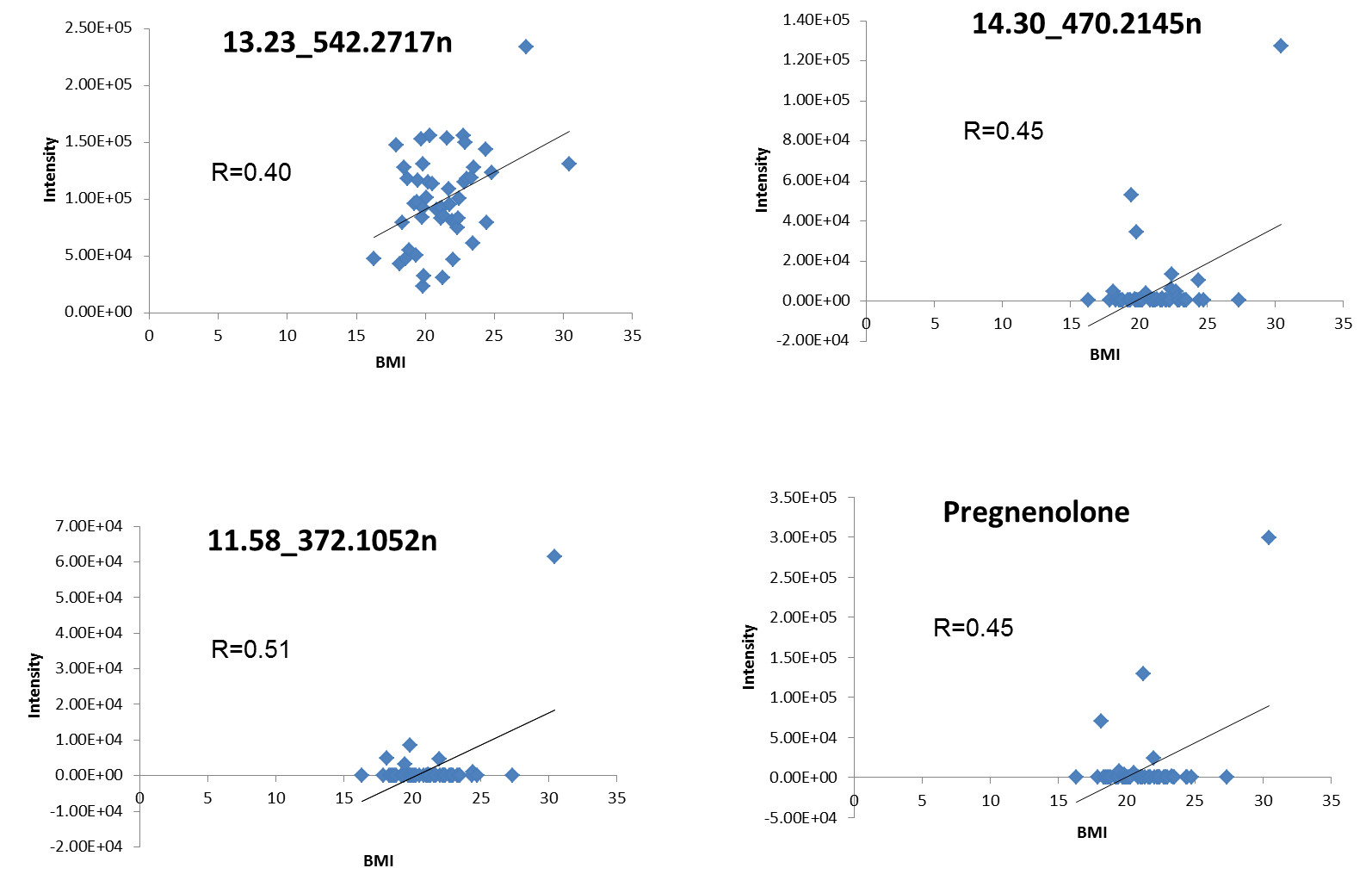


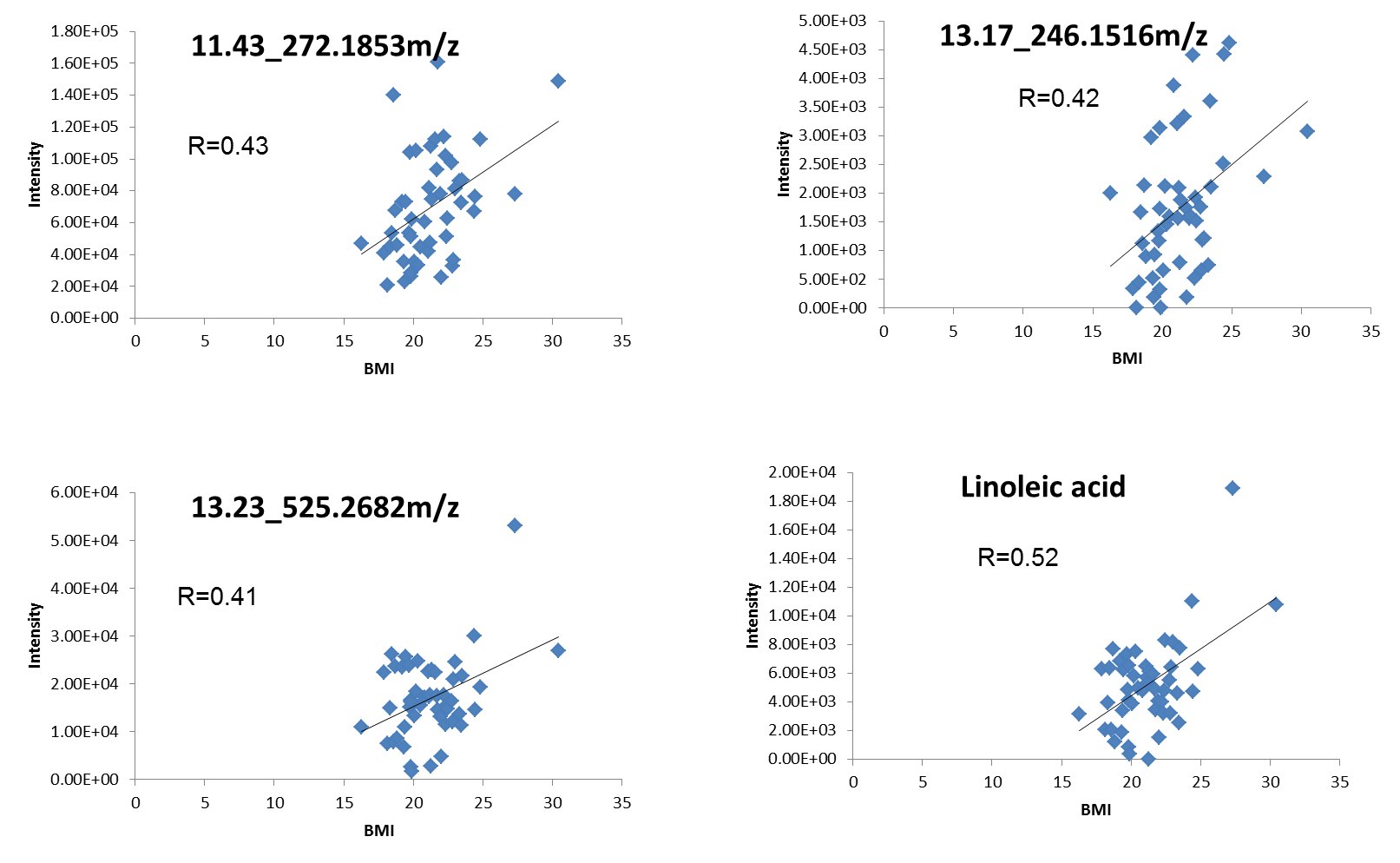


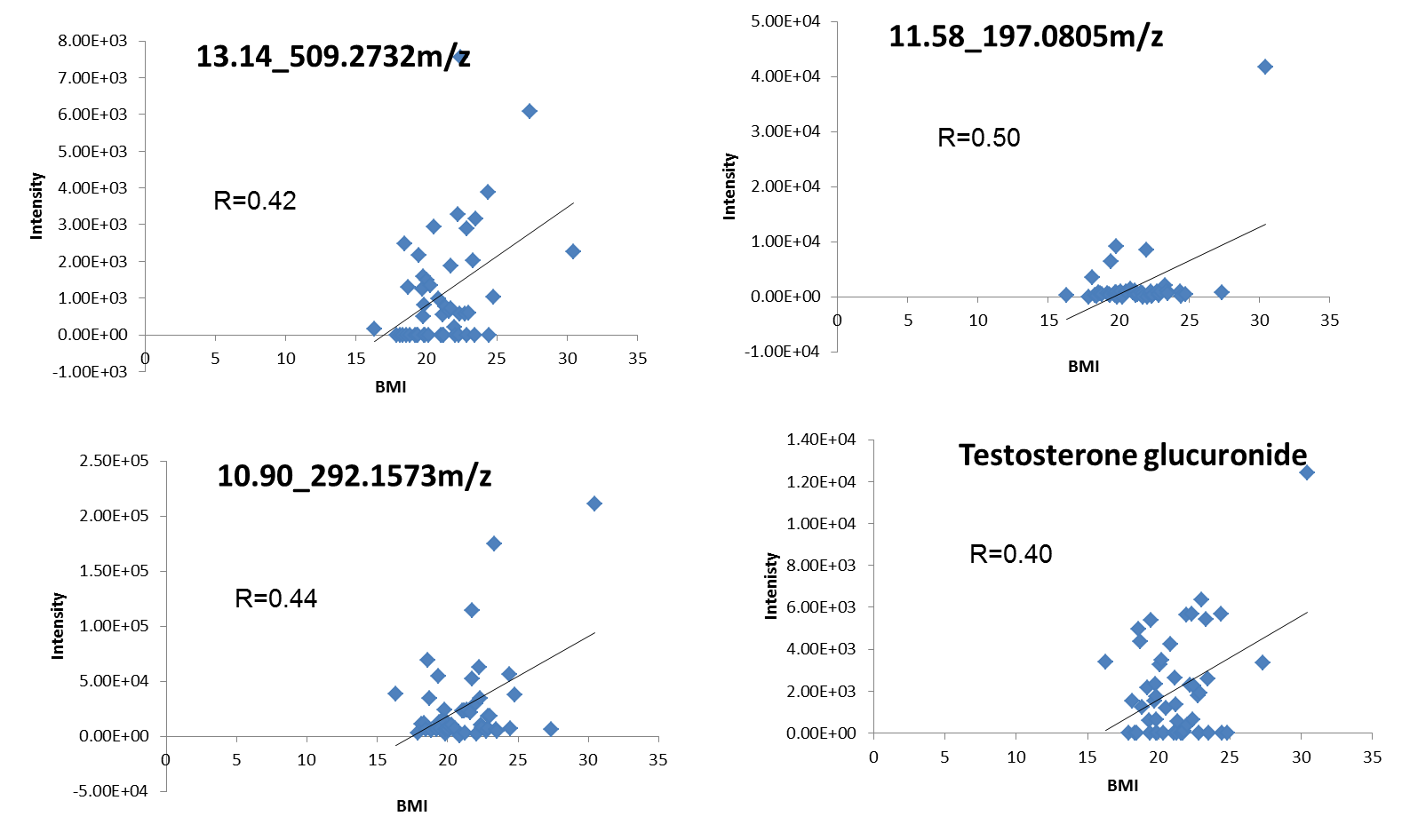


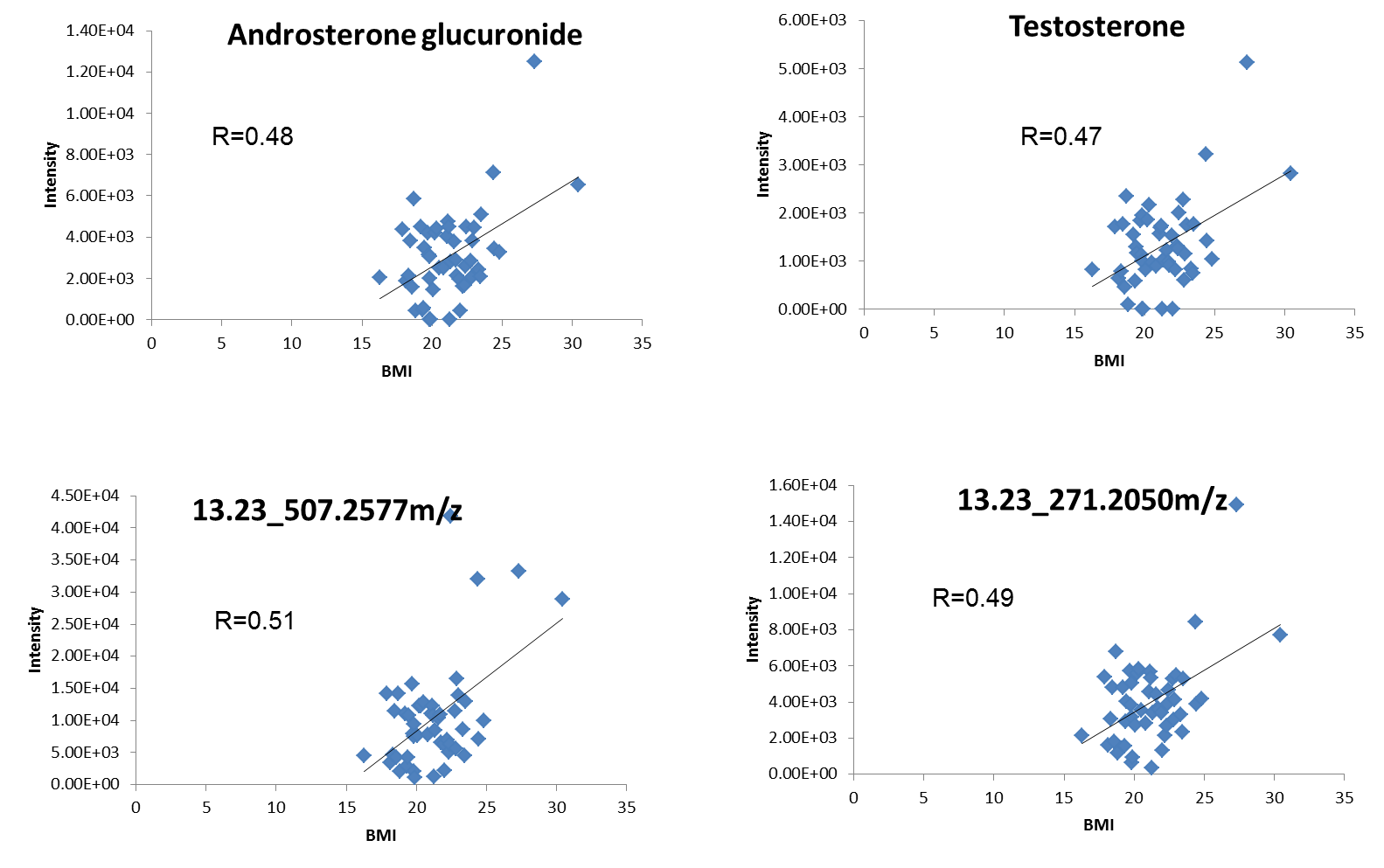


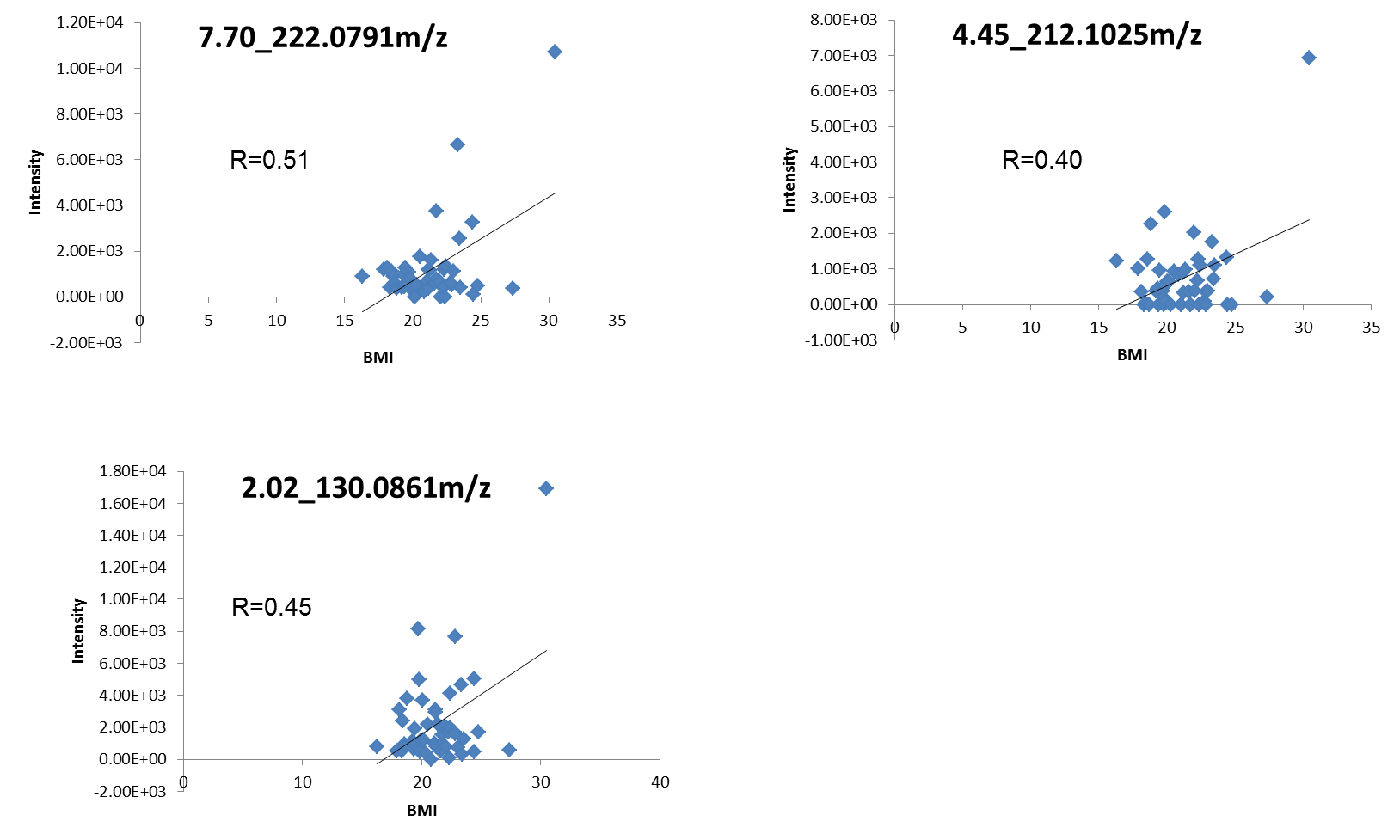


**Fig. S4** PCA analysis of metabolic profiling between the control and GDM in the three trimesters. a. Score plot of metabolic profiling in the first trimester between the control and GDM. b. Score plot of metabolic profiling in the second trimester between the control and GDM. c. Score plot of metabolic profiling in the third trimester between the control and GDM


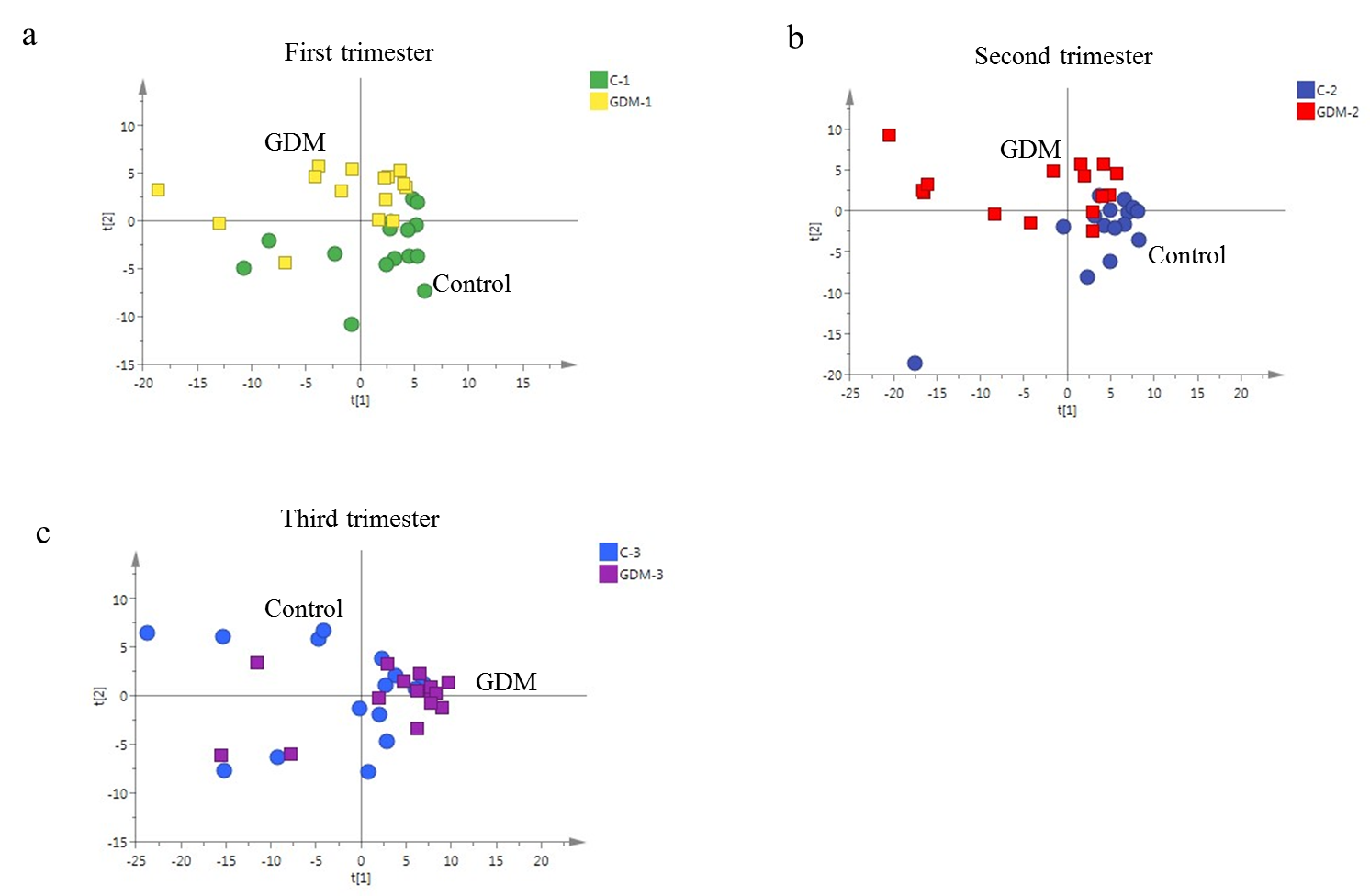


**Fig. S5** Analysis of metabolic profiling discrimination between the control and GDM during the three trimesters. a. ROC plots based on urine metabolic profiling to quantify the discrimination degree of the first trimester between control and GDM. b. ROC plots based on urine metabolic profiling to quantify the discrimination degree of the second trimester between control and GDM. c. ROC plots based on urine metabolic profiling to quantify the discrimination degree of the third trimester between control and GDM.


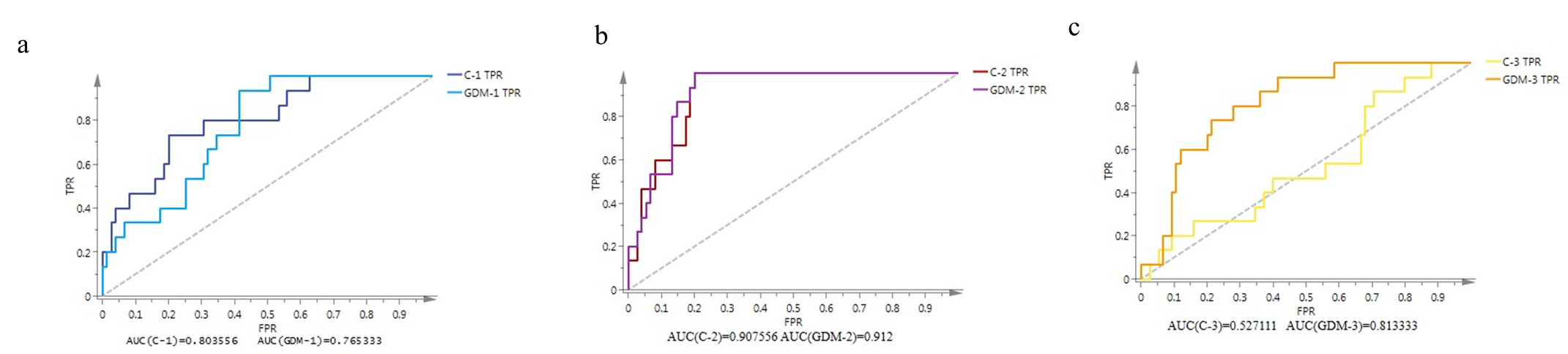


**Fig.S6** PCA score plot of metabolic profiling in the first trimester between the control and SA.


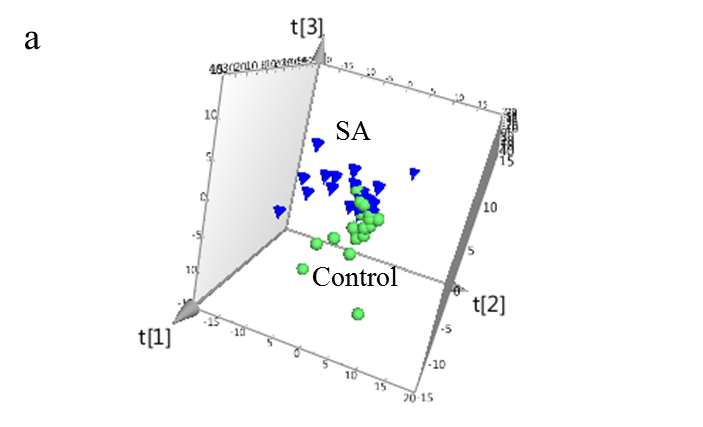


**Fig S7 MS2 spectra of the putatively annotated metabolites extracted by Progenesis QI**

**
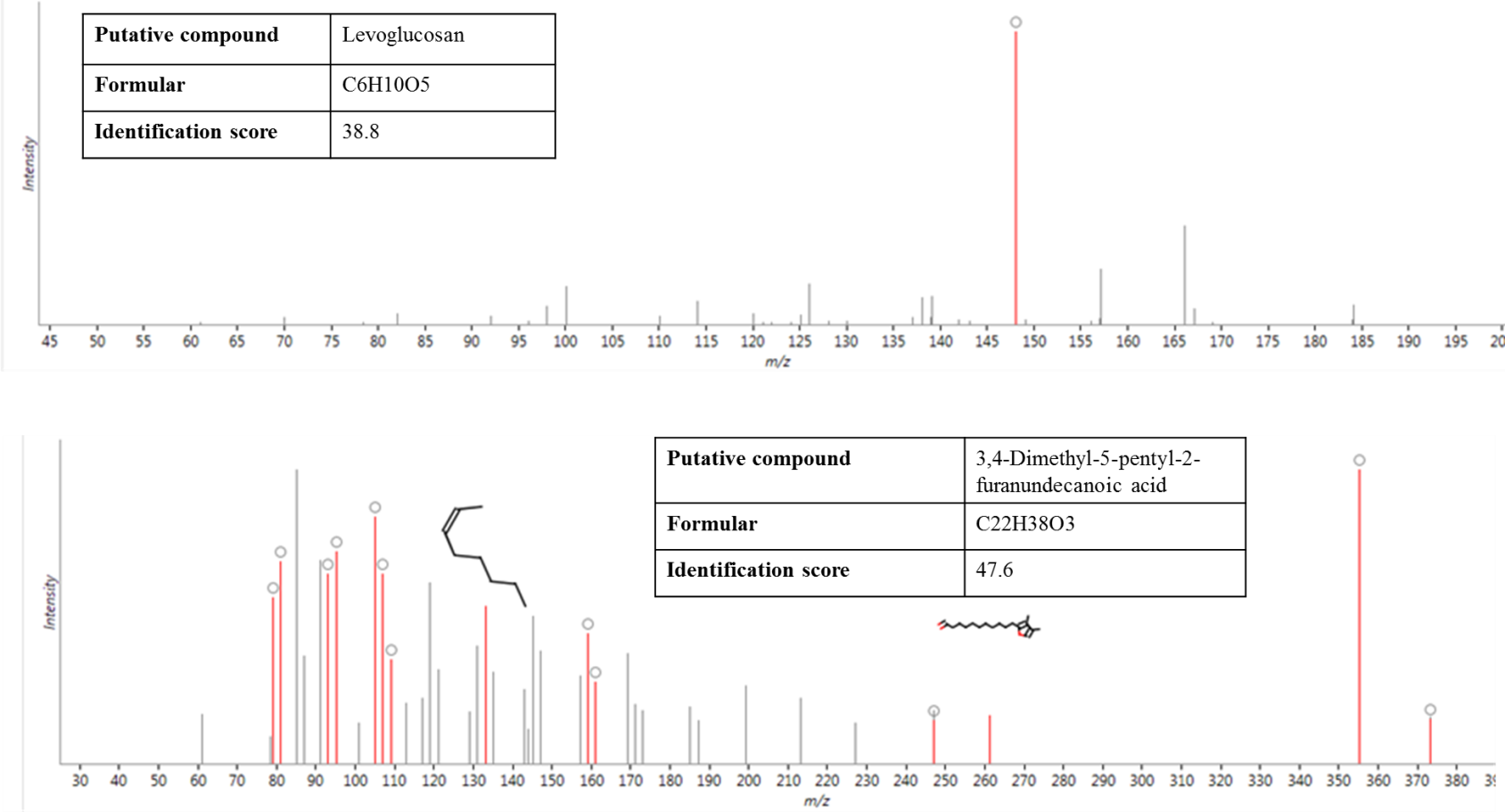
**

**
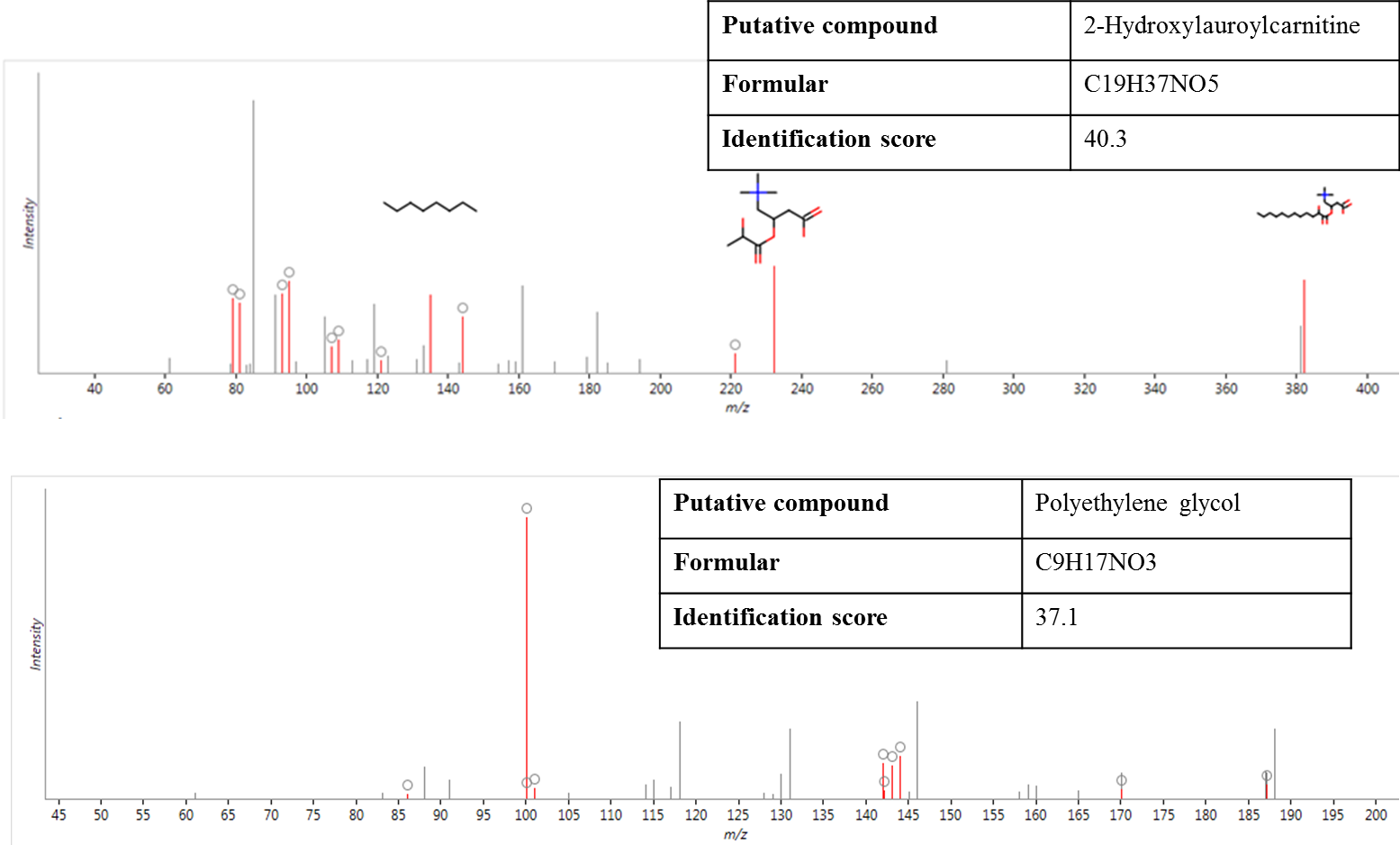
**

**
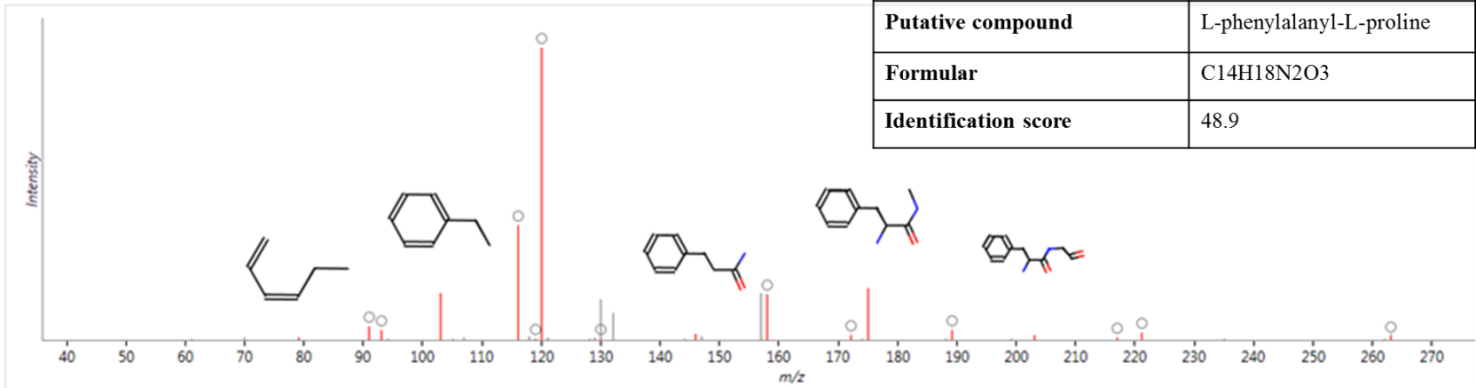
**

**
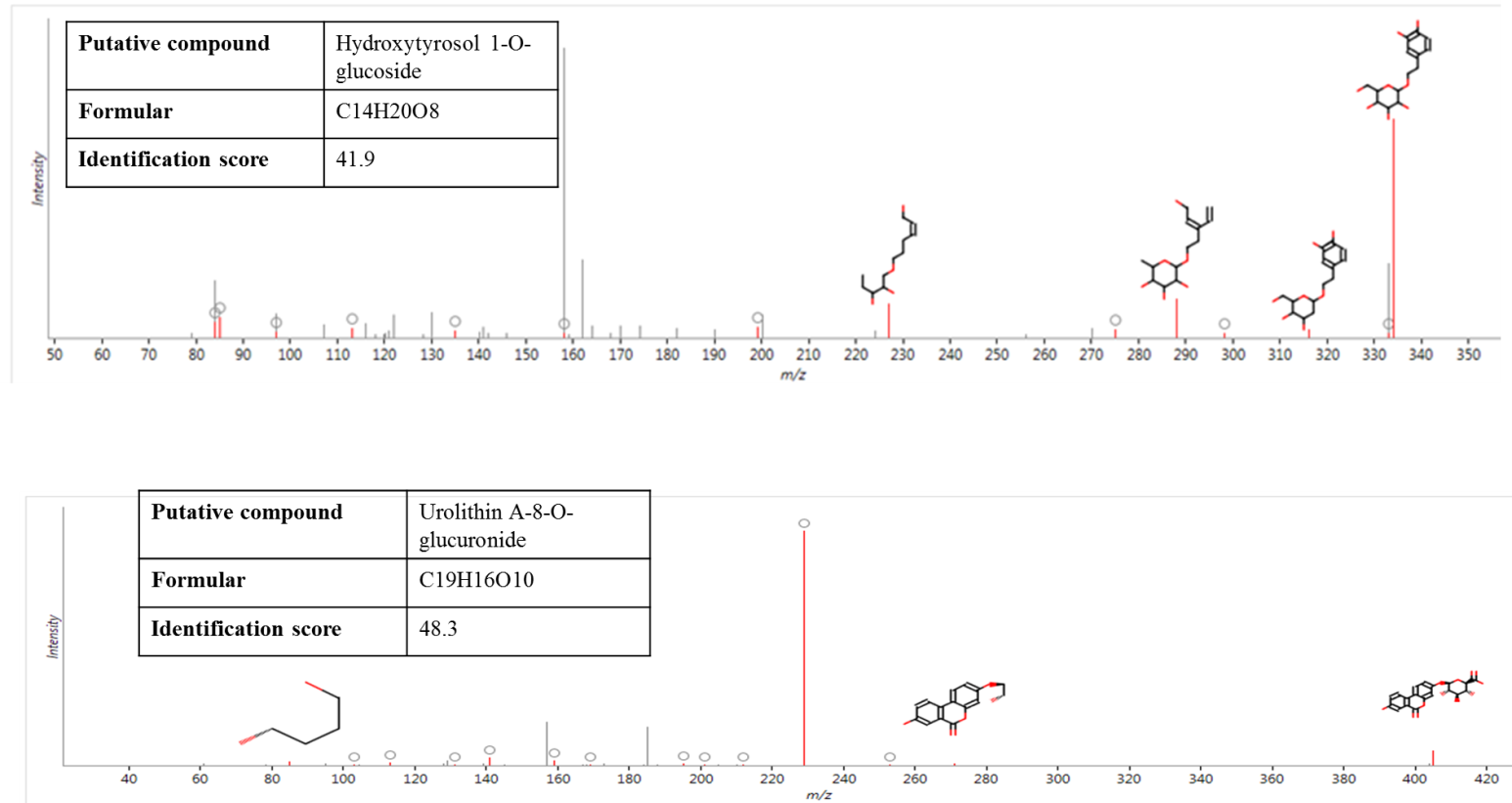
**

**
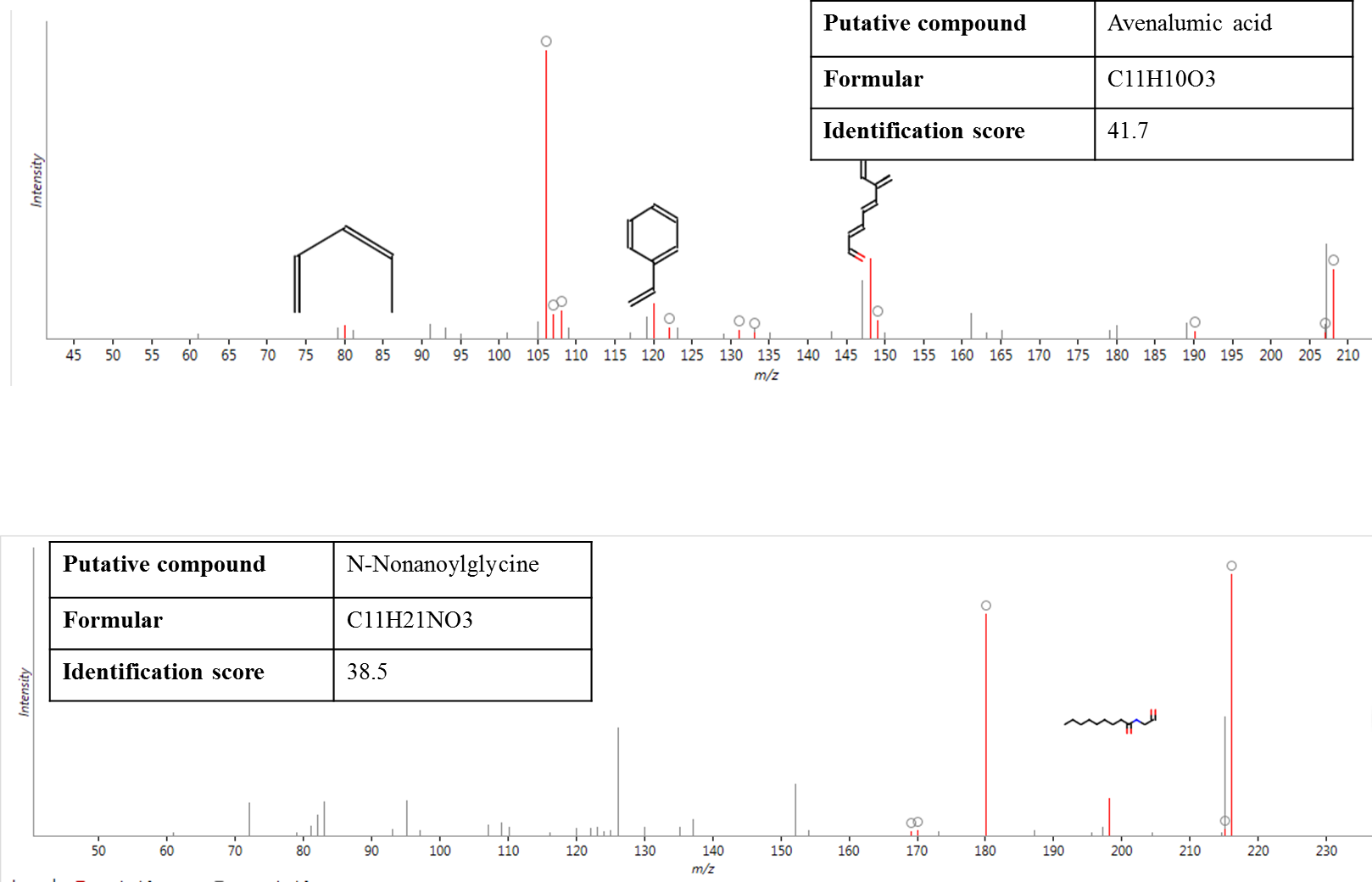
**

**
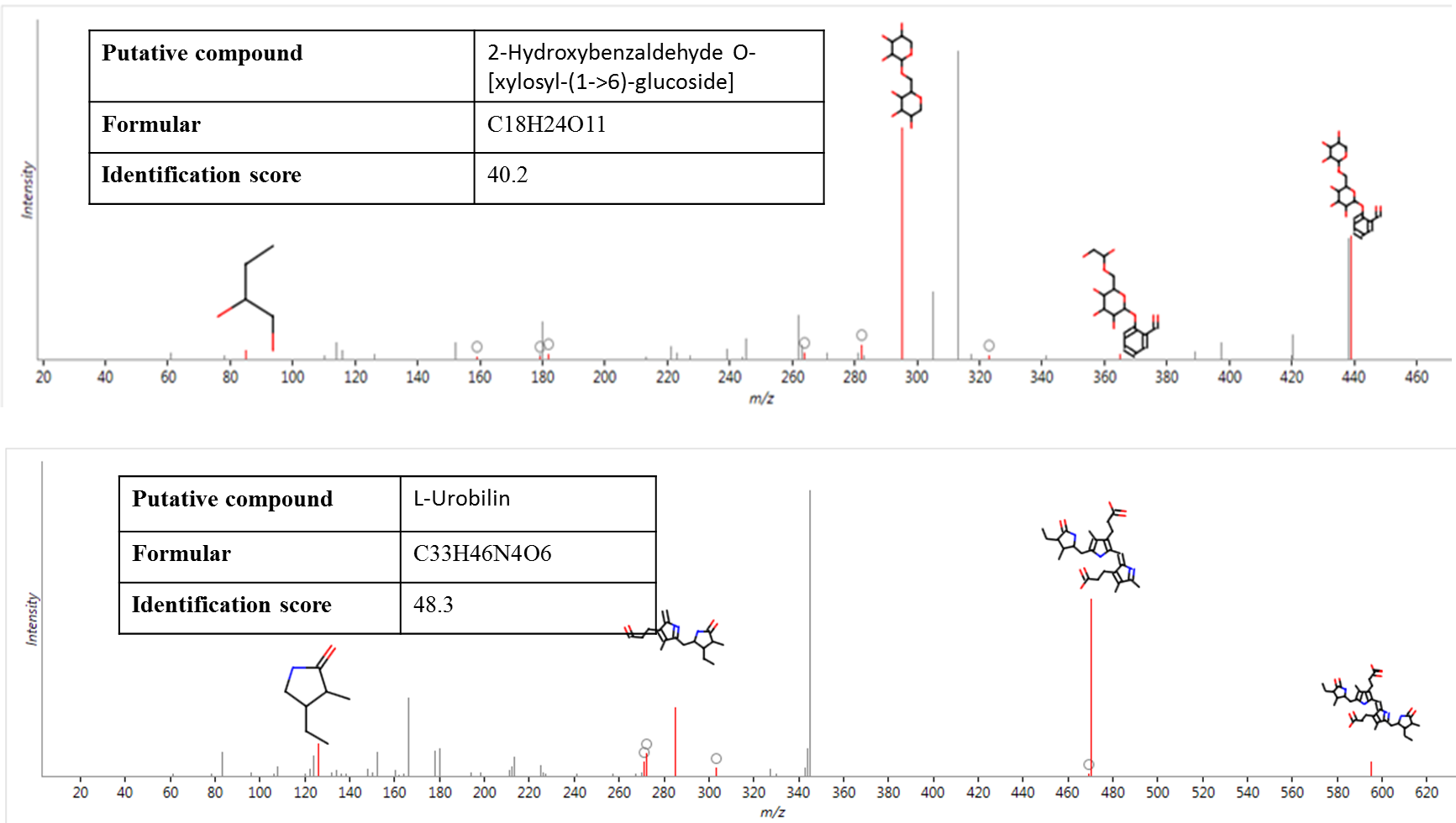
**

**
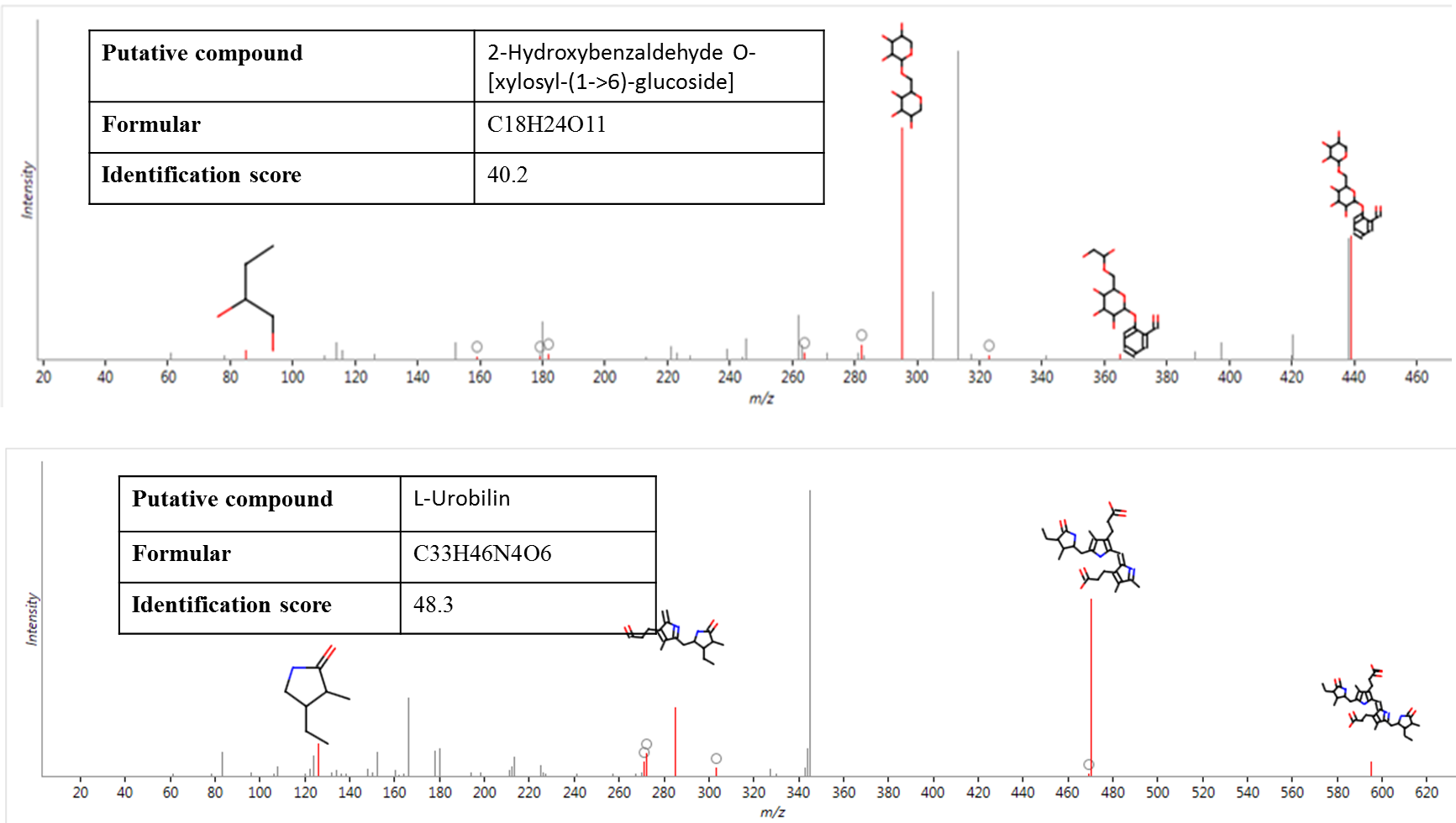
**

**
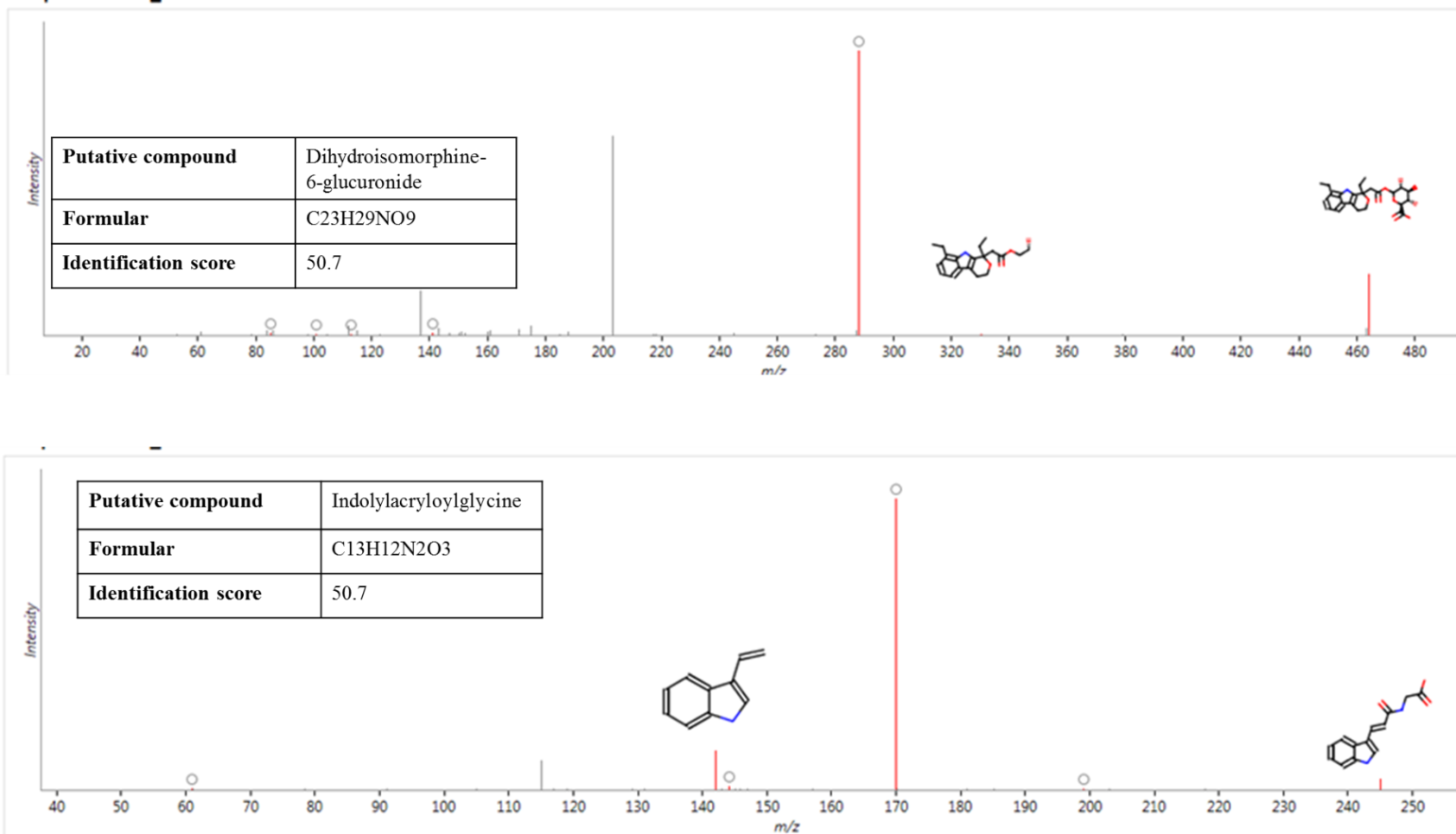
**

**
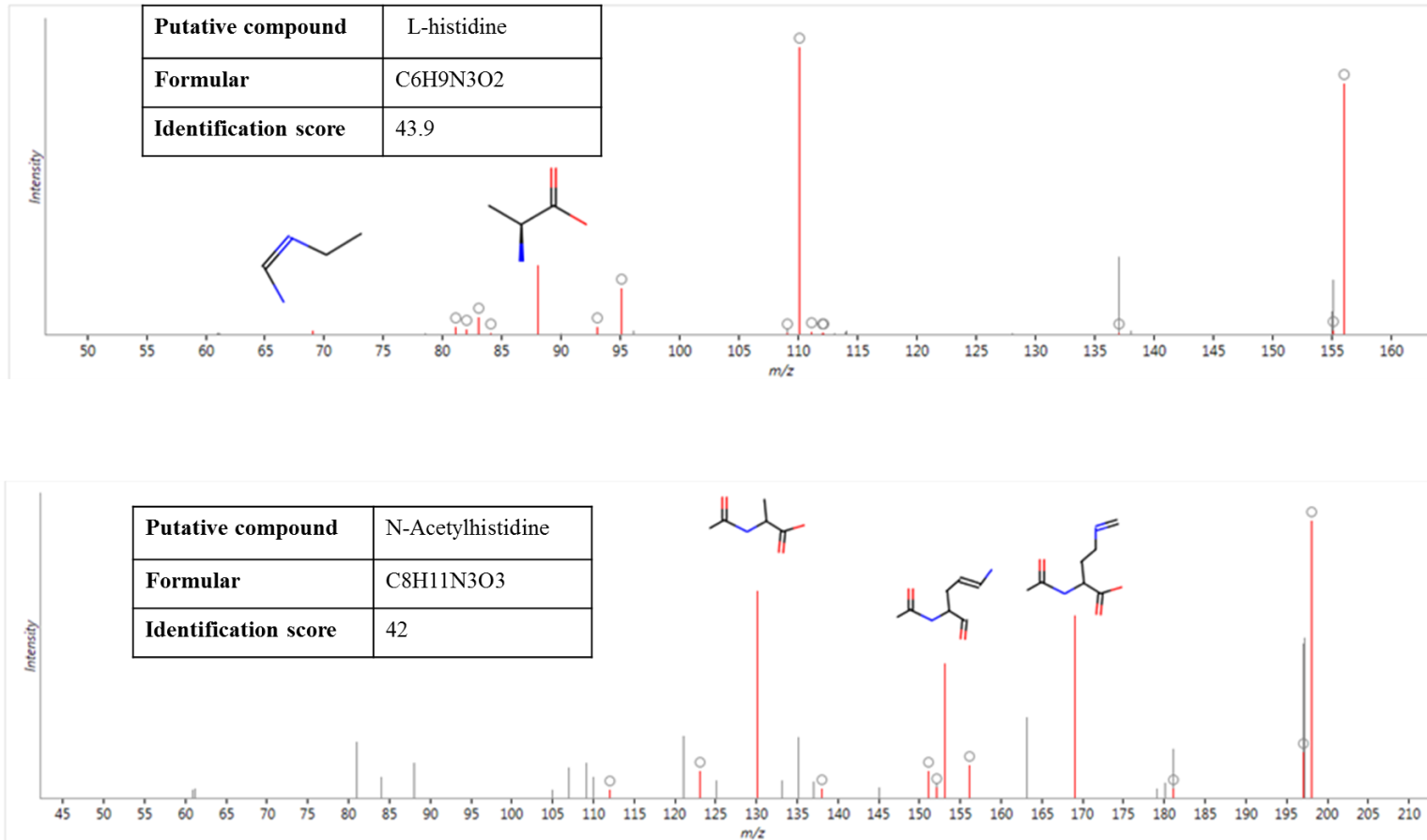
**

**
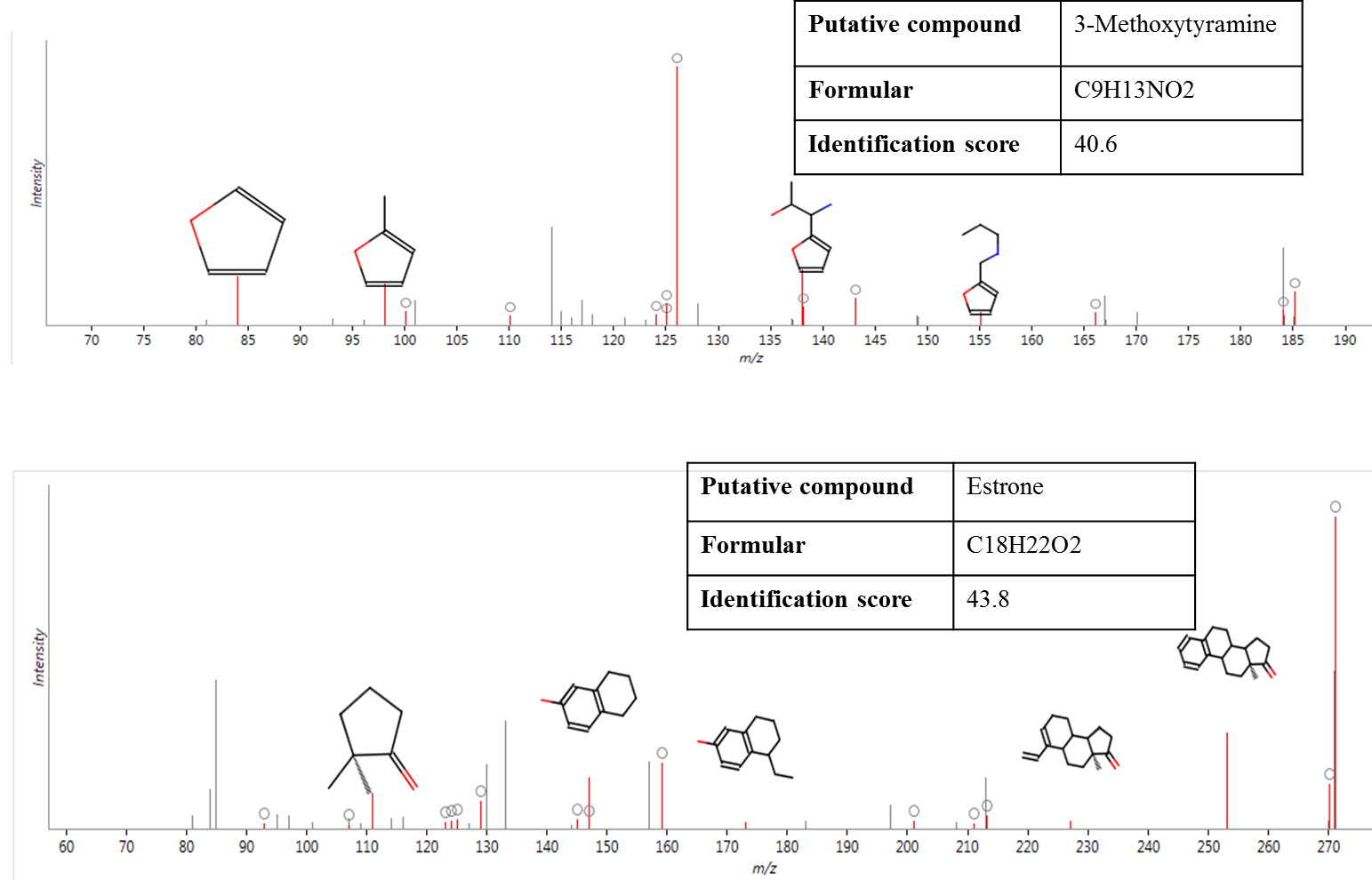
**

**
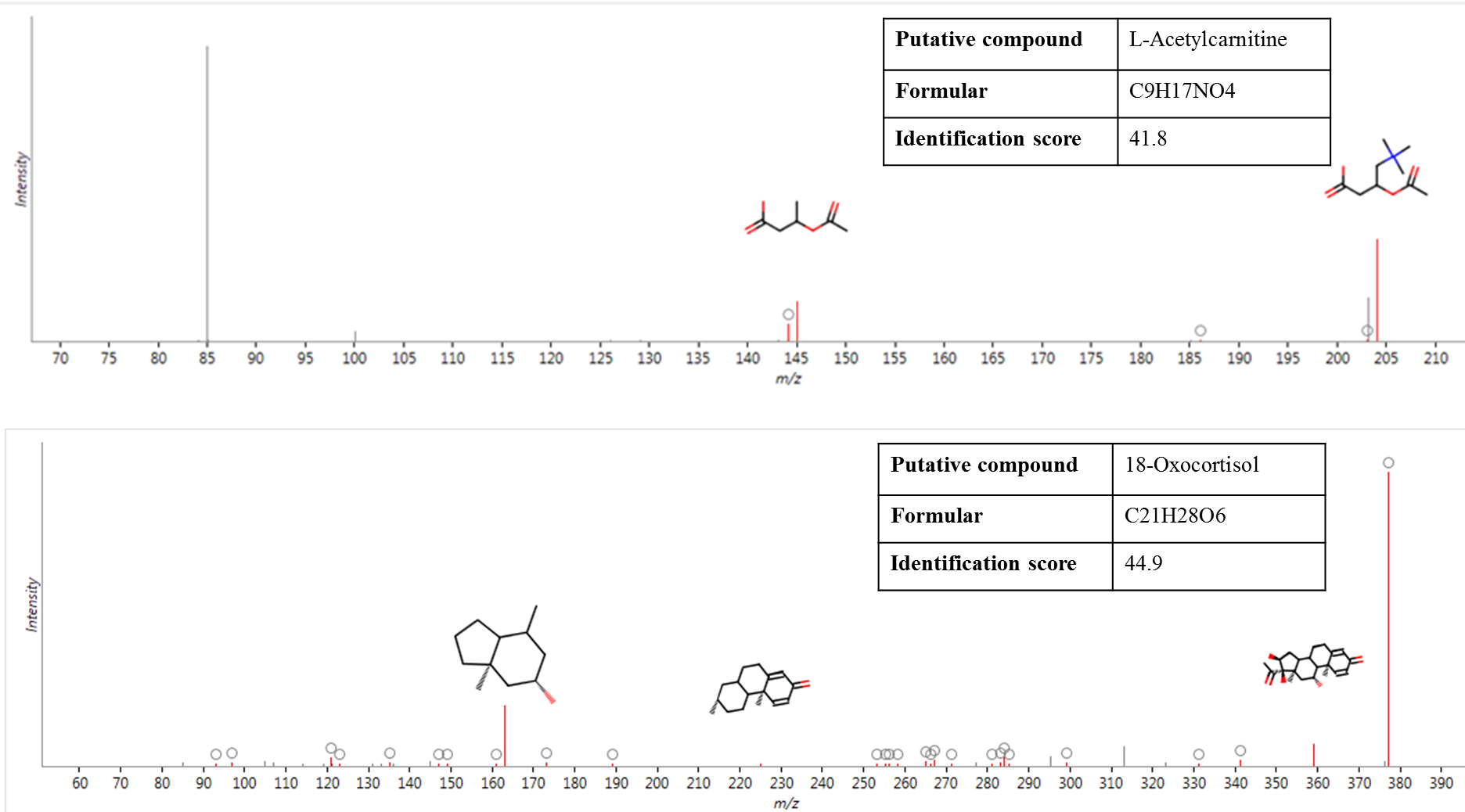
**

**
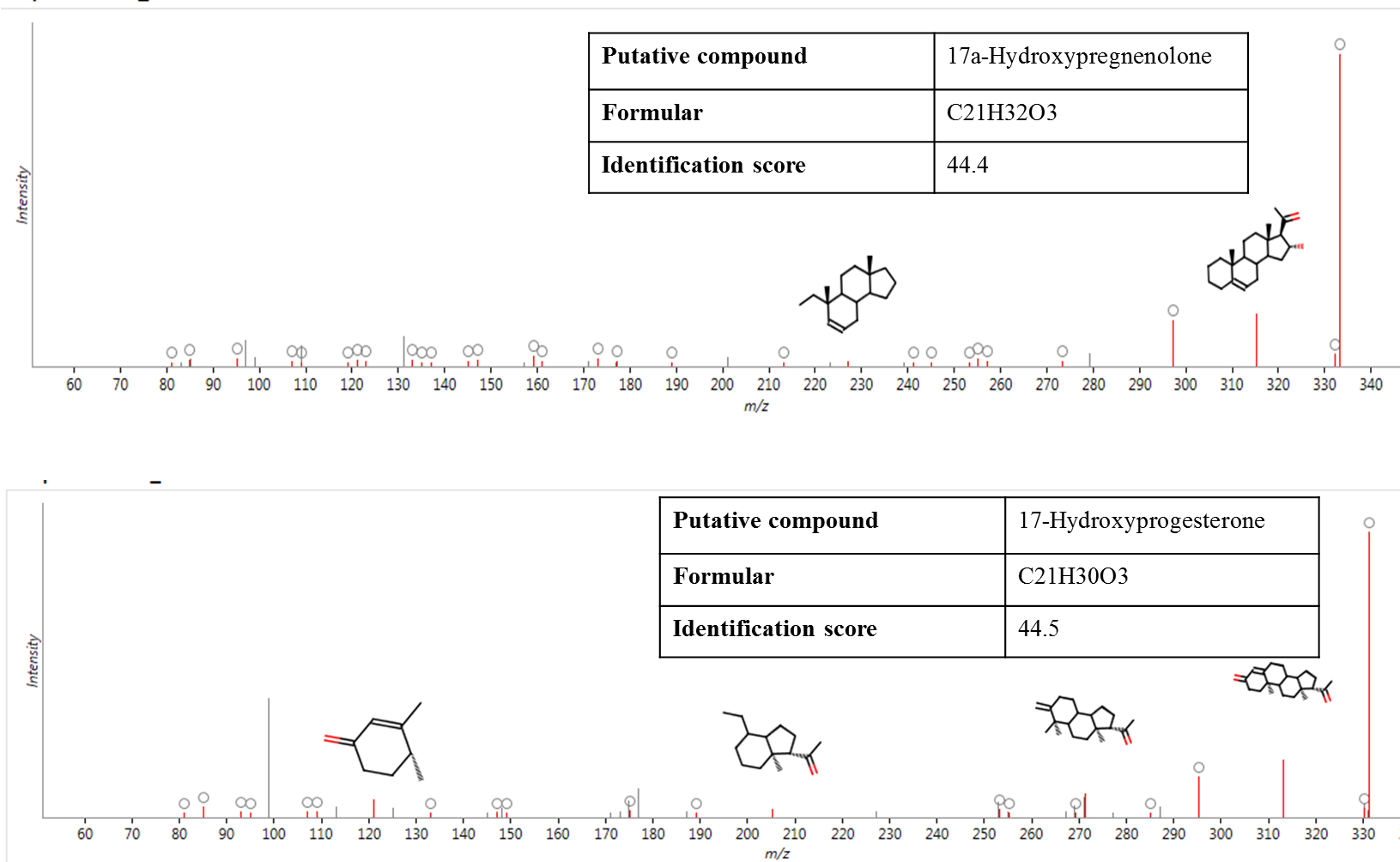
**

**
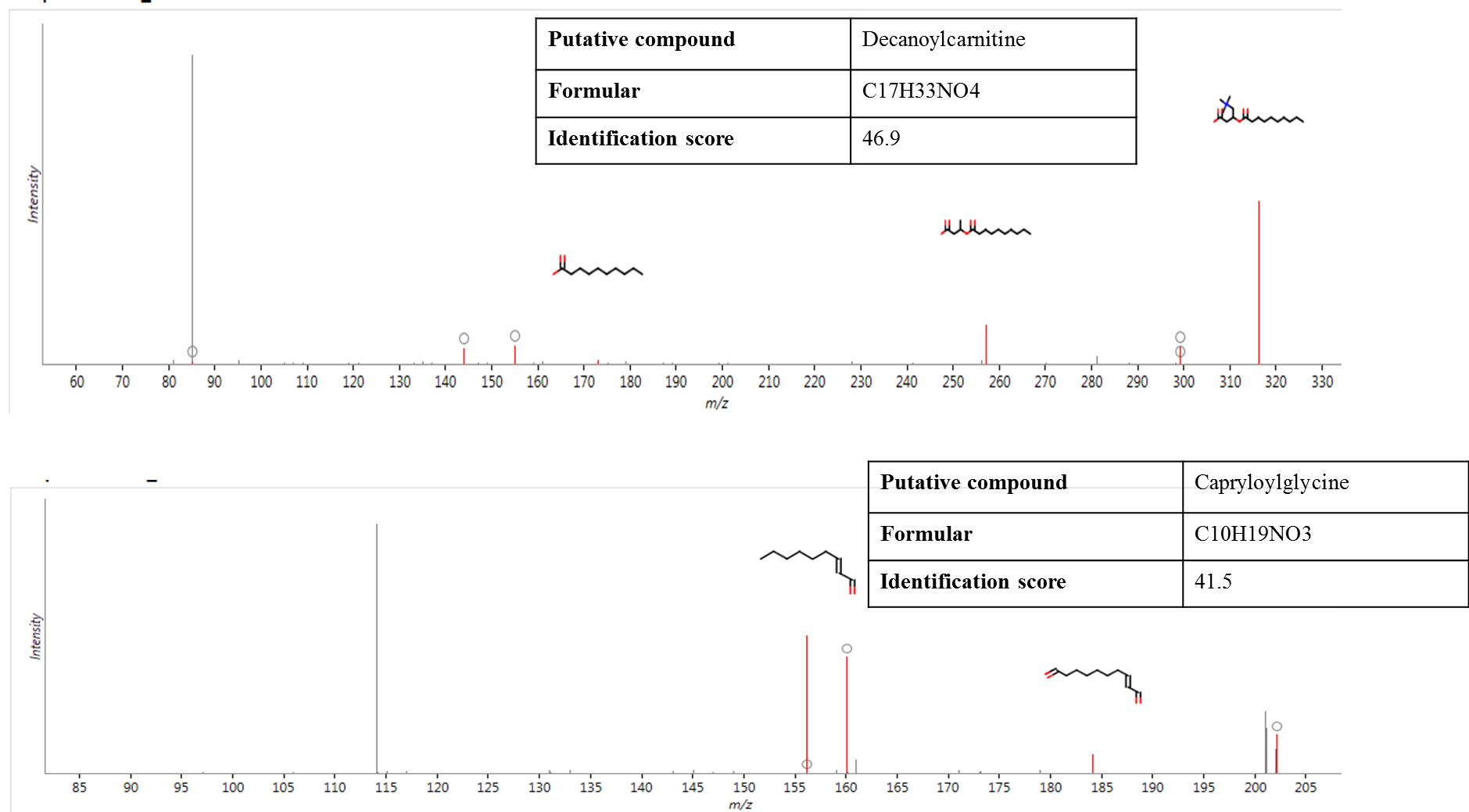
**

**
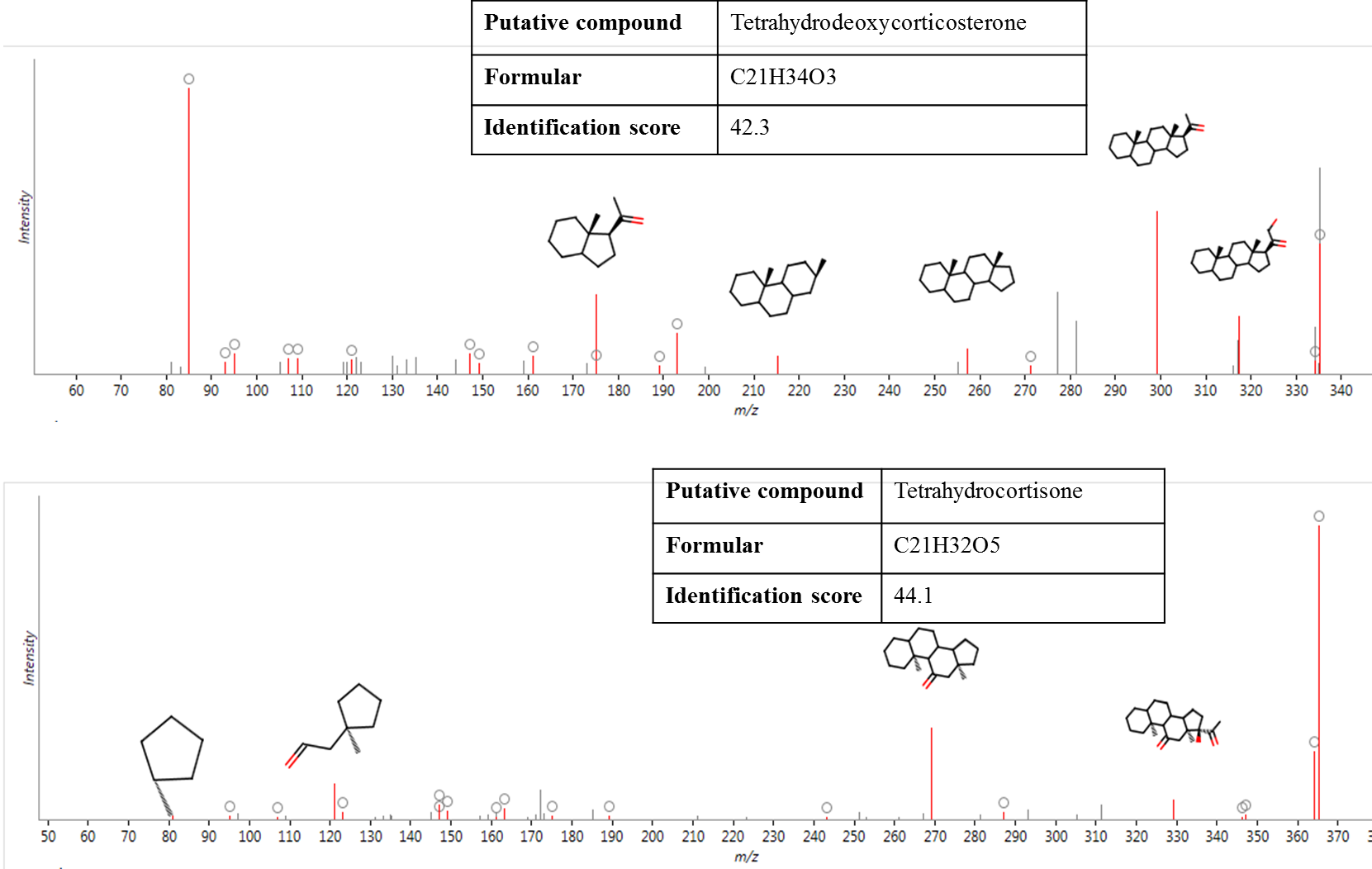
**

**
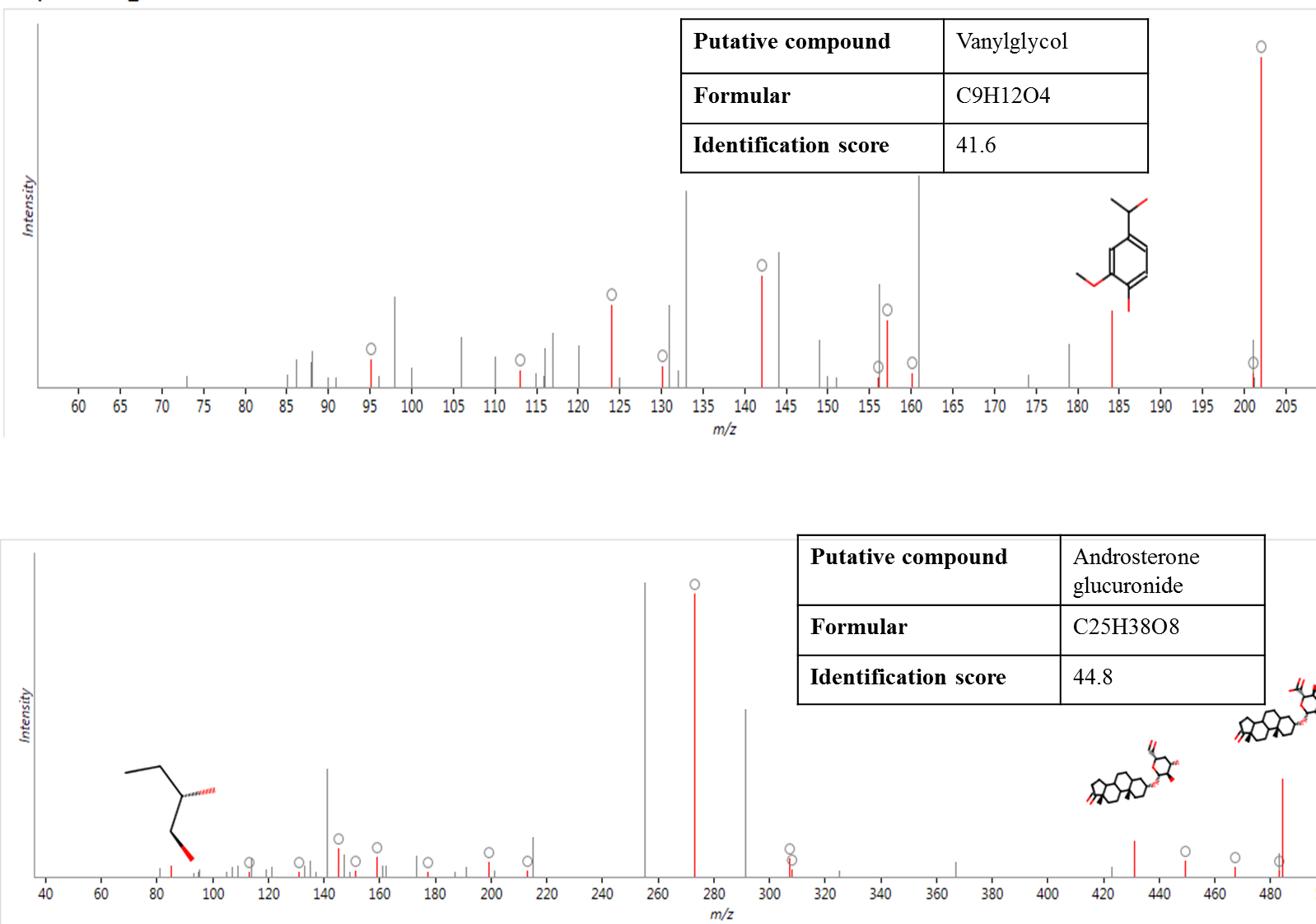
**

**
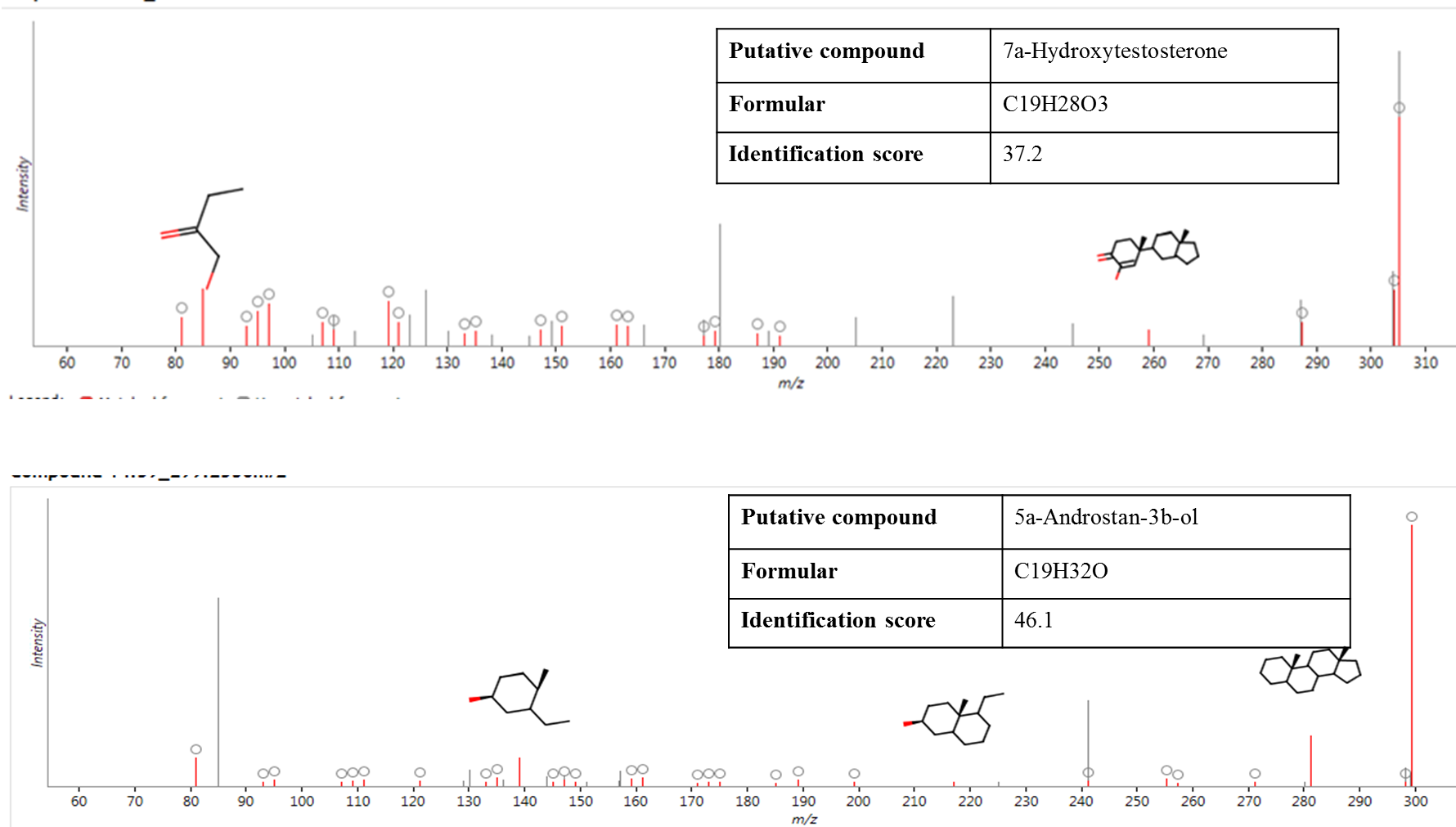
**

**
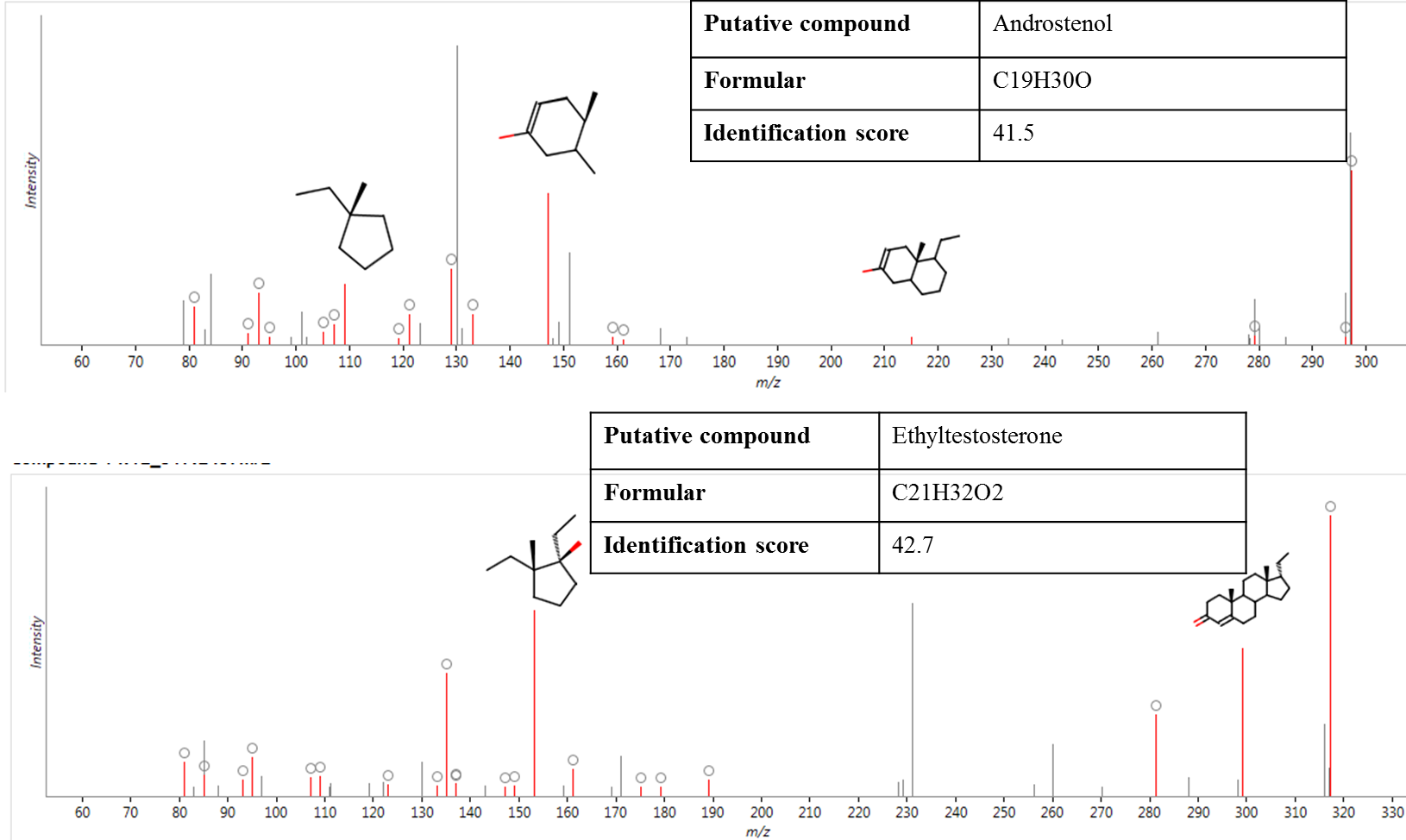
**

**
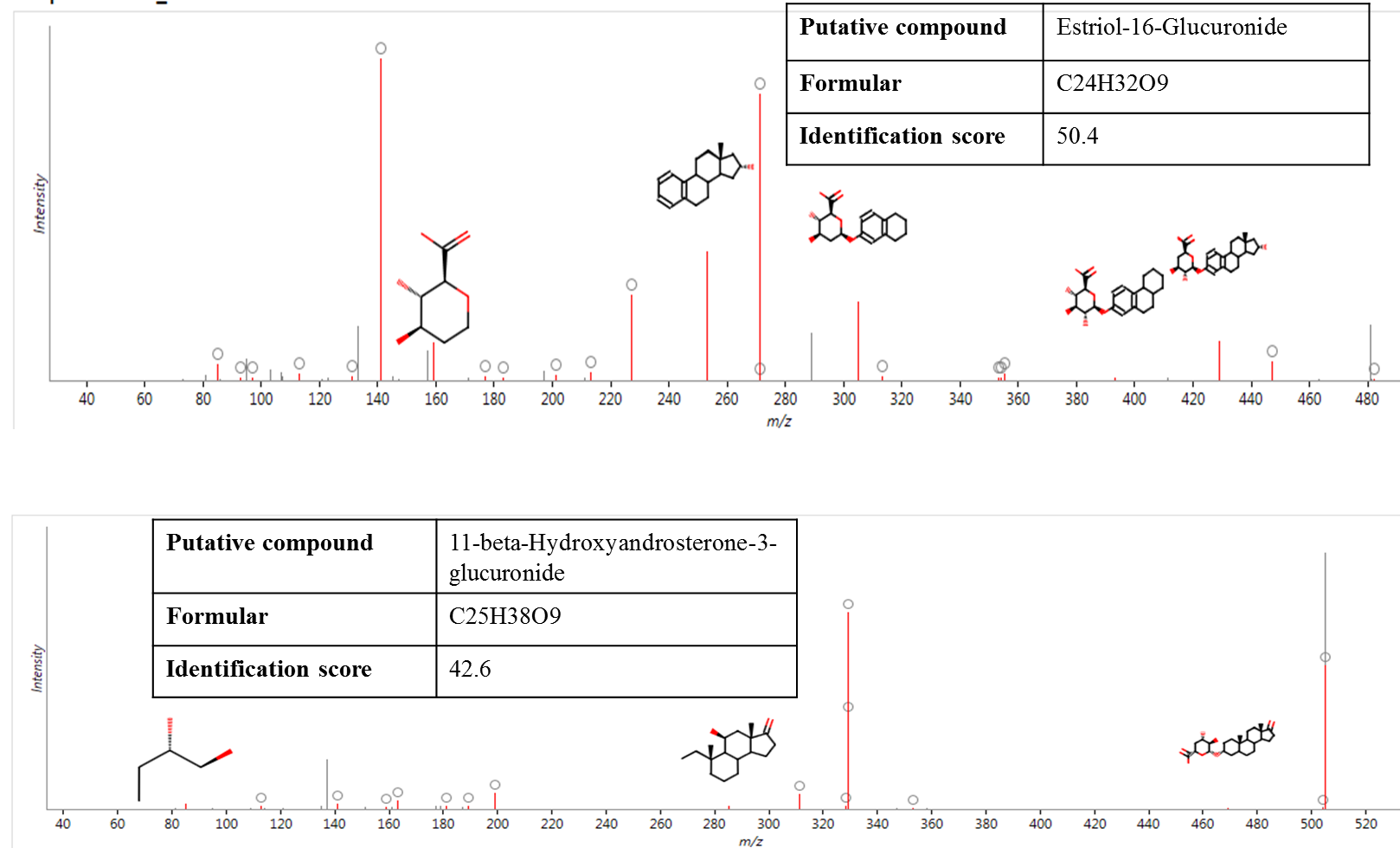
**

**
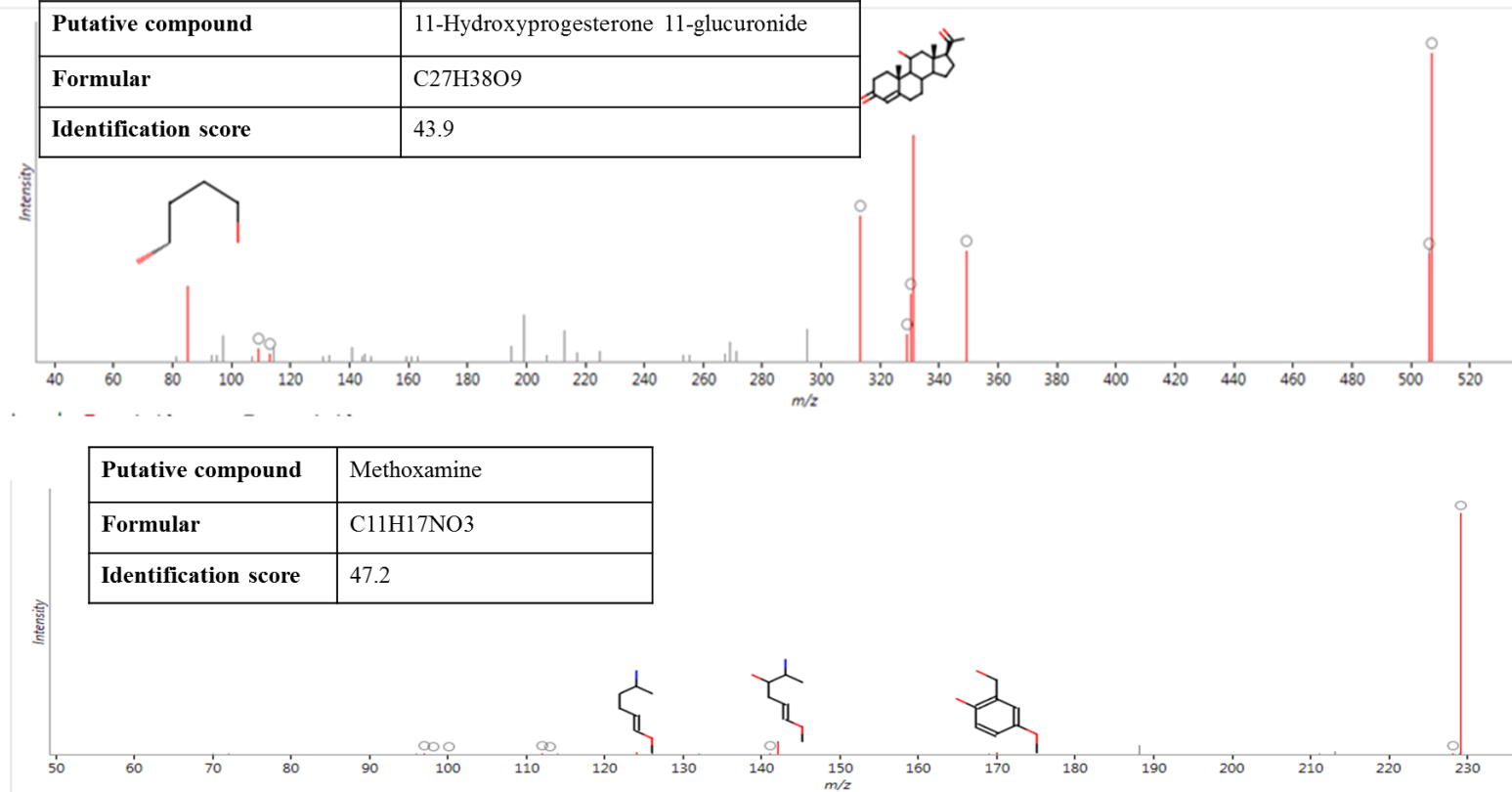
**

**
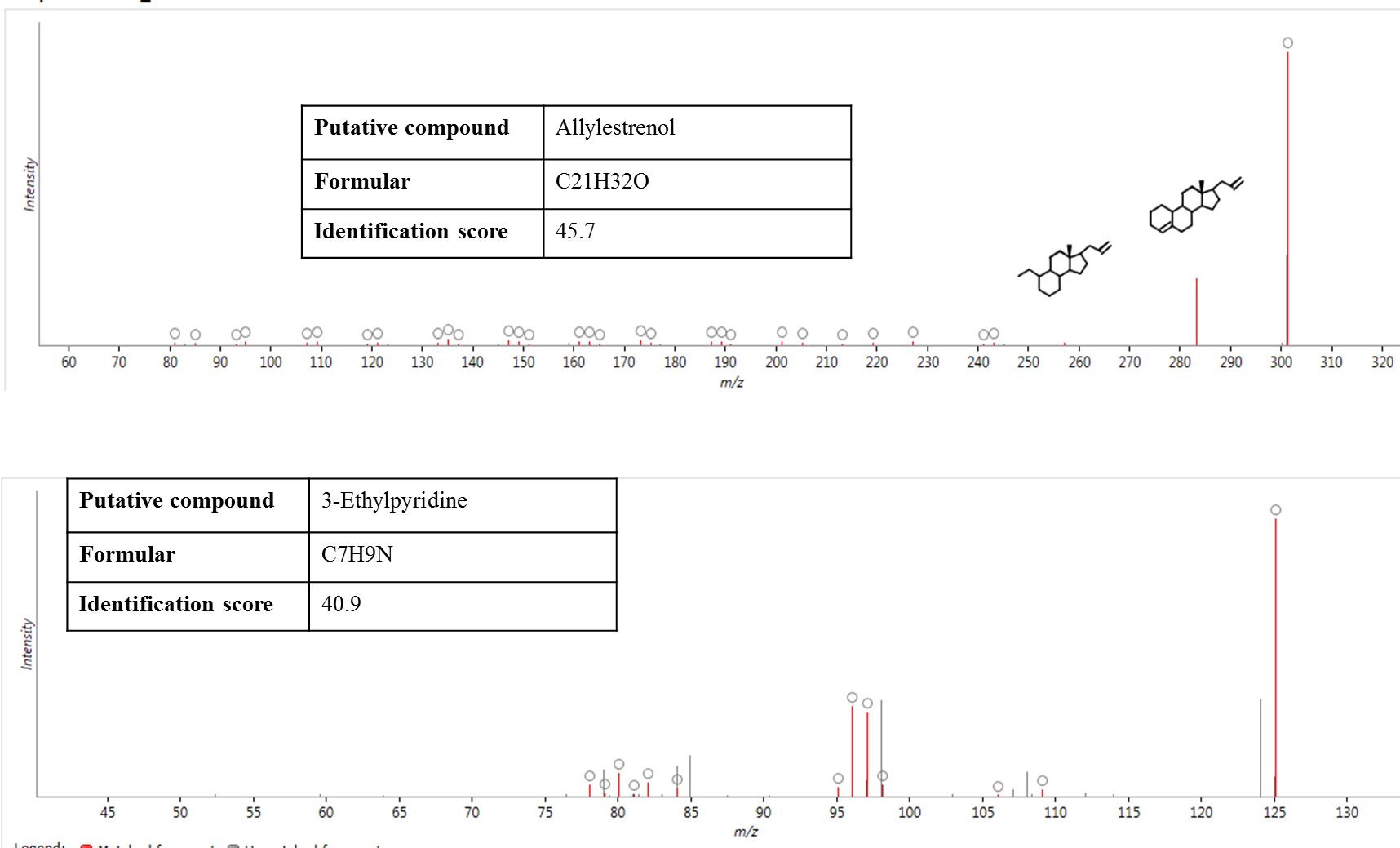
**

**
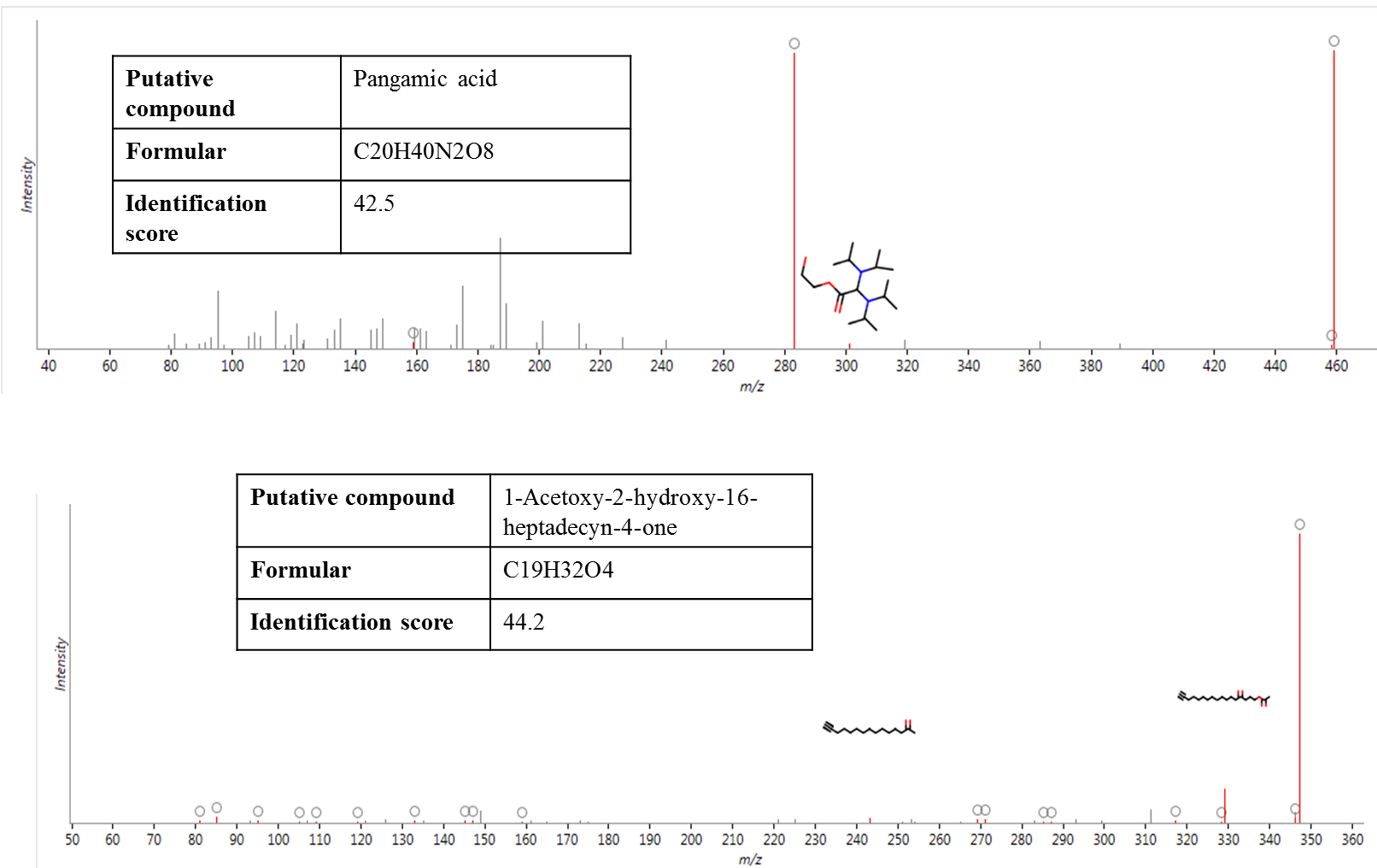
**

**
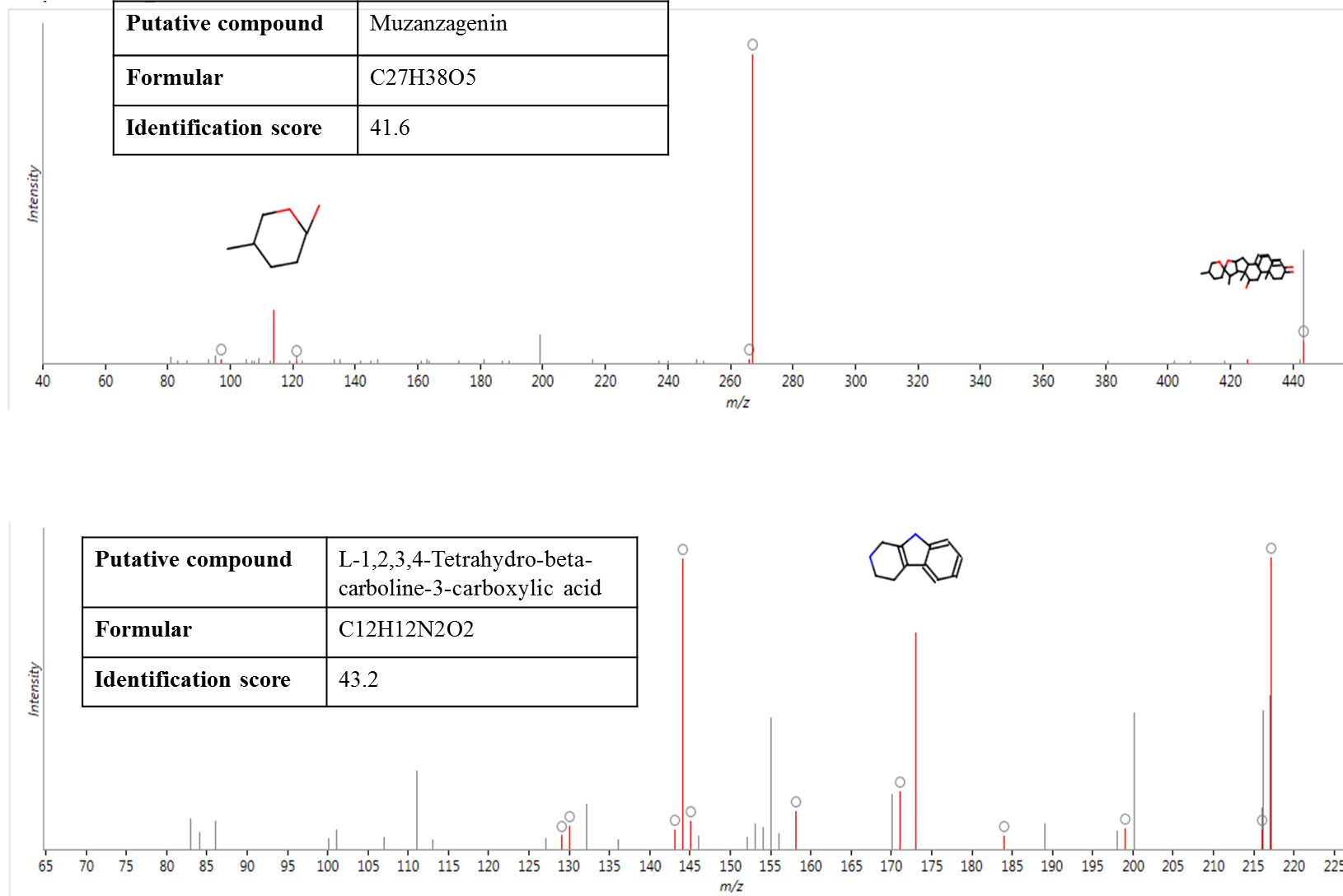
**

**
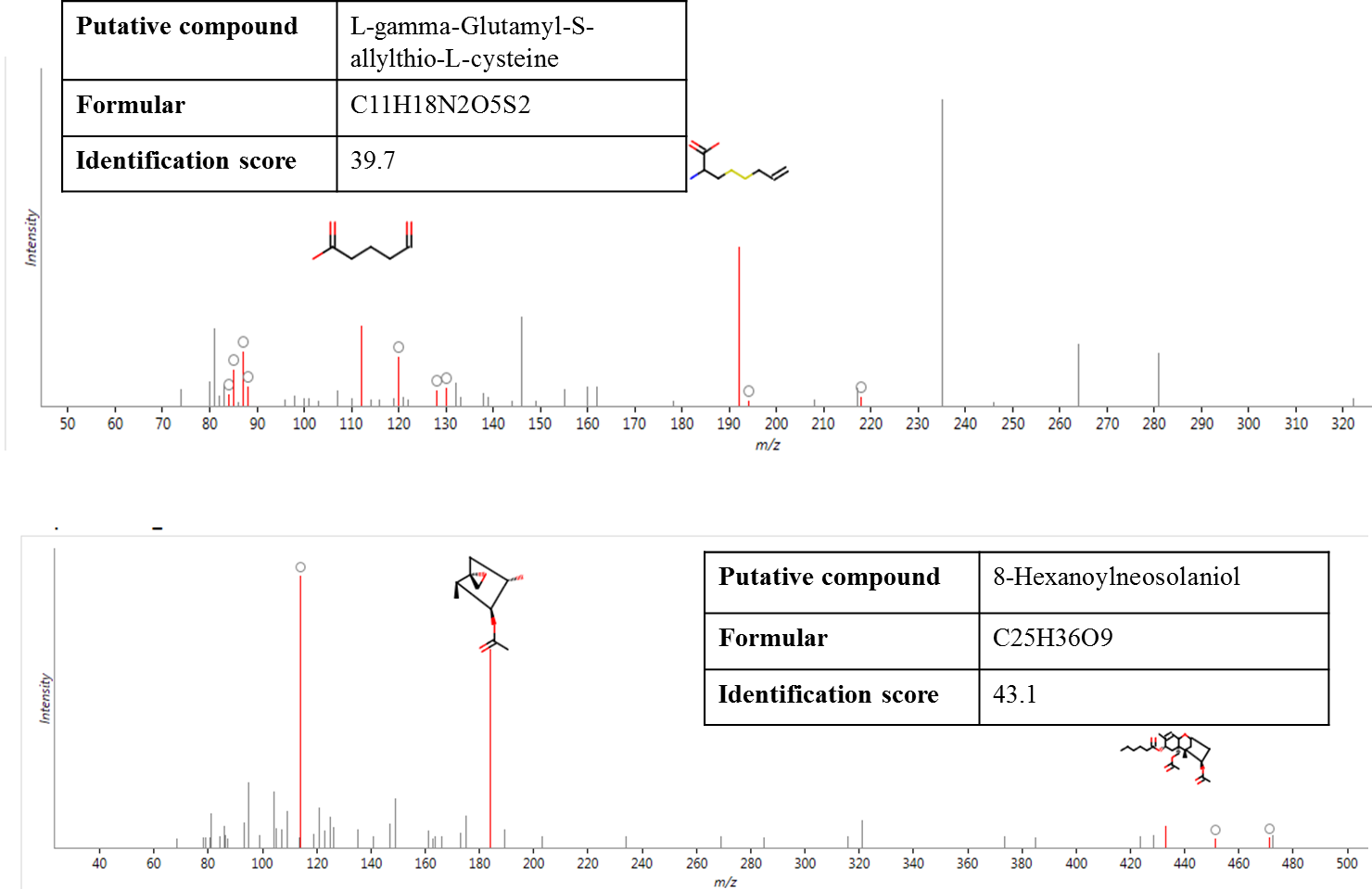
**

**
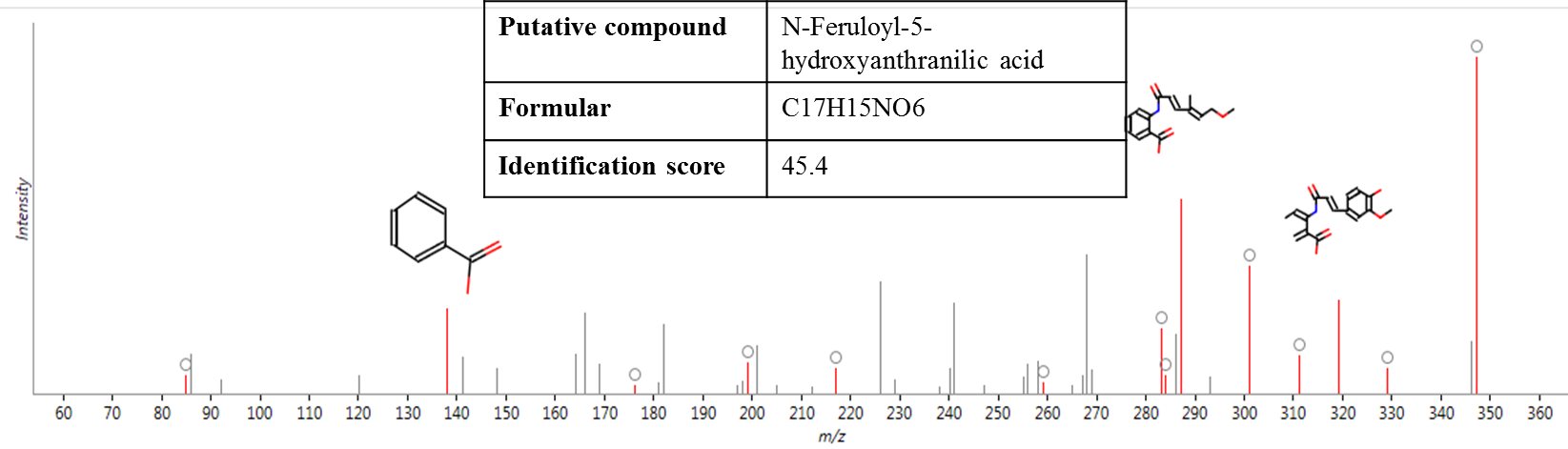
**

**Table S1** Differential metabolites during health pregnancy progression

| Compound | Compound ID | Description | Formula | Mass error | isotope pattern, | Score | Change trend | p-value |
| --- | --- | --- | --- | --- | --- | --- | --- | --- |
| 10.51_347.1232m/z | HMDB38575 | (Z)-N-Feruloyl-5-hydroxyanthranilic acid | C17H15NO6 | 2.10 | 94.81 | 45.4 | up | 3.65E-06 |
| 12.20_376.2689m/z | HMDB12983 | Kinetensin 1-3 | C15H30N6O4 | 0.05 | 94.78 | 42.5 | Down | 2.07E-03 |
| 12.37_271.1685m/z | HMDB00145 | Estrone | C18H22O2 | -3.72 | 93.59 | 43.8 | up | 2.37E-03 |
| 12.37_429.1901m/z | HMDB41091 | 1-(4-Hydroxy-3,5-dimethoxyphenyl)-7-(4-hydroxy-3-methoxyphenyl)-3,5-heptanediol | C22H30O7 | 2.10 | 95.17 | 41.4 | up | 8.15E-07 |
| 12.37_447.2003m/z | HMDB04483 | Estrone glucuronide | C24H30O8 | -1.74 | 91.13 | 41.8 | up | 2.66E-07 |
| 12.37_464.2039n | HMDB06766 | Estriol-16-Glucuronide | C24H32O9 | 0.00 | 91.14 | 47.2 | up | 1.82E-05 |
| 12.73_297.2206m/z | HMDB05935 | Androstenol | C19H30O | -1.70 | 95.24 | 41.5 | up | 5.99E-08 |
| 13.59_347.2210m/z | HMDB31007 | 1-Acetoxy-2-hydroxy-16-heptadecyn-4-one | C19H32O4 | 0.15 | 94.36 | 44.2 | Down | 4.61E-02 |
| 13.59_365.2316m/z | HMDB00903 | Tetrahydrocortisone | C21H32O5 | -2.95 | 93.64 | 44.1 | Down | 2.27E-02 |
| 13.71_299.2364m/z | HMDB05830 | 5a-Androstan-3b-ol | C19H32O | -1.71 | 94.77 | 46.1 | up | 6.08E-08 |
| 13.73_317.2468m/z | HMDB06002 | Ethyltestosterone | C21H32O2 | -0.01 | 93.88 | 42.7 | up | 6.67E-40 |
| 13.73_377.1952m/z | HMDB00332 | 18-Oxocortisol | C21H28O6 | -3.71 | 93.02 | 44.9 | up | 1.55E-14 |
| 13.78_305.2103m/z | HMDB03956 | 7alpha-Hydroxytestosterone | C19H28O3 | -1.74 | 80.67 | 37.2 | up | 5.42E-13 |
| 13.85_314.2320m/z | HMDB13205 | 9-Decenoylcarnitine | C17H31NO4 | 0.05 | 96.89 | 48.1 | Down | 2.72E-02 |
| 13.92_506.2717n | HMDB38327 | (3b,9R)-5-Megastigmene-3,9-diol 9-[apiosyl-(1->6)-glucoside] | C24H42O11 | 0.16 | 88.66 | 41.1 | up | 1.43E-06 |
| 13.98_316.1481m/z | HMDB00651 | Decanoylcarnitine | C17H33NO4 | -3.69 | 92.83 | 46.9 | up | 2.42E-06 |
| 14.08_507.2569m/z | HMDB10364 | 11-Hydroxyprogesterone 11-glucuronide | C27H38O9 | 0.03 | 90.84 | 43.9 | up | 7.33E-25 |
| 14.27_201.1361n | HMDB00832 | Capryloylglycine | C10H19NO3 | -3.69 | 98.87 | 41.5 | Down | 4.75E-02 |
| 14.29_333.2418m/z | HMDB00363 | 17a-Hydroxypregnenolone | C21H32O3 | -3.70 | 92.65 | 44.4 | up | 1.49E-04 |
| 14.40_459.2733m/z | HMDB29949 | Pangamic acid | C20H40N2O8 | 0.12 | 94.68 | 42.5 | up | 3.44E-32 |
| 14.57_505.2410m/z | HMDB10351 | 11-beta-Hydroxyandrosterone-3-glucuronide | C25H38O9 | 0.00 | 92.73 | 42.6 | up | 1.39E-18 |
| 14.72_331.2262m/z | HMDB00374 | 17-Hydroxyprogesterone | C21H30O3 | -3.69 | 94.30 | 44.5 | up | 1.98E-28 |
| 14.92_301.2520m/z | HMDB15500 | Allylestrenol | C21H32O | 0.11 | 94.45 | 45.7 | up | 3.42E-14 |
| 15.19_335.2575m/z | HMDB00879 | Tetrahydrodeoxycorticosterone | C21H34O3 | -3.69 | 94.99 | 42.3 | up | 3.95E-15 |
| 15.48_466.2560n | HMDB02829 | Androsterone glucuronide | C25H38O8 | -2.94 | 92.20 | 44.8 | Down | 1.06E-02 |
| 16.04_443.2784m/z | HMDB32601 | Muzanzagenin | C27H38O5 | 0.15 | 92.28 | 41.6 | up | 1.38E-06 |
| 16.04_498.2731m/z | HMDB38559 | 8-Hexanoylneosolaniol | C25H36O9 | 2.10 | 85.84 | 43.1 | up | 7.65E-06 |
| 2.13_204.1227m/z | HMDB00201 | L-Acetylcarnitine | C9H17NO4 | -3.72 | 97.96 | 41.8 | Down | 4.22E-02 |
| 2.49_229.1544m/z | HMDB14861 | Methoxamine | C11H17NO3 | 0.09 | 97.78 | 47.2 | Down | 4.70E-08 |
| 5.16_232.1076m/z | HMDB41809 | 4-(Methylnitrosamino)-1-(3-pyridyl)-1-butanol | C10H15N3O2 | 2.10 | 88.30 | 39.7 | Down | 8.17E-26 |
| 7.57_395.1424n | HMDB14502 | Mefloquine | C17H16F6N2O | 0.09 | 90.96 | 40 | Down | 3.94E-04 |
| 7.61_413.1233m/z | HMDB30565 | (Z)-Resveratrol 4'-glucoside | C20H22O8 | 0.13 | 91.51 | 44.5 | Down | 3.95E-02 |
| 8.09_202.1071m/z | HMDB01490 | Vanylglycol | C9H12O4 | -2.95 | 95.04 | 41.6 | Down | 3.46E-02 |
| 8.11_299.0910m/z | HMDB41520 | 5-De-O-methyltoddanol | C15H16O5 | 2.10 | 91.68 | 41 | Down | 3.58E-03 |
| 8.63_125.0839n | HMDB02055 | o-Cresol | C7H8O | -2.95 | 92.45 | 38.6 | Down | 3.32E-02 |
| 8.63_125.1072m/z | HMDB29734 | 3-Ethylpyridine | C7H9N | 0.12 | 95.59 | 40.9 | Down | 3.47E-02 |
| 8.63_184.1210n | HMDB00022 | 3-Methoxytyramine | C9H13NO2 | -3.72 | 89.92 | 40.6 | Down | 1.12E-05 |
| 8.96_322.0653n | HMDB38515 | L-gamma-Glutamyl-S-allylthio-L-cysteine | C11H18N2O5S2 | 0.16 | 89.53 | 39.7 | Down | 1.36E-12 |
| 9.18_217.0969m/z | HMDB35665 | L-1,2,3,4-Tetrahydro-beta-carboline-3-carboxylic acid | C12H12N2O2 | 0.15 | 94.19 | 43.2 | Down | 1.67E-21 |

**Table S2** Information of seven potential biomarkers in the first trimester for GDM prediction

| **Metabolite biomarker** | Compound ID | Formula | Mass error | Isotope pattern | Score | **AUC** | ***p*-value** | **Log2 FC** |
| --- | --- | --- | --- | --- | --- | --- | --- | --- |
| Levoglucosan | HMDB00640 | C6H10O5 | -1.73509 | 94.64141 | 38.8 | 0.84 (0.68-0.95) | 0.005 | 1.57 |
| 1'-Acetoxyeugenol acetate | HMDB36544 | C14H16O5 | -1.75974 | 94.04719 | 46 | 0.81 (0.64-0.96) | 0.02 | -1.02 |
| 3,4-Dimethyl-5-pentyl-2-furanundecanoic acid | HMDB31126 | C22H38O3 | 4.581747 | 92.8313 | 47.6 | 0.79 (0.61-0.94) | 0.003 | -1.95 |
| 2-Hydroxylauroylcarnitine | HMDB13164 | C19H37NO5 | 4.971845 | 91.74722 | 40.3 | 0.78 (0.59-0.94) | 0.03 | -1.11 |
| Polyethylene glycol | HMDB37790 | C9H17NO3 | -2.42287 | 92.24819 | 37.1 | 0.76 (0.59-0.91) | 0.04 | 3.04 |
| L-phenylalanyl-L-proline | HMDB11177 | C14H18N2O3 | -1.37813 | 92.47587 | 48.9 | 0.76 (0.58-0.91) | 0.02 | -1.07 |
| 6-Hydroxy-5-methoxyindole glucuronide | HMDB10362 | C15H17NO8 | -1.53447 | 93.08648 | 53.7 | 0.70 (0.51-0.86) | 0.02 | 2.85 |

**Table S3** Differential metabolites in SA

| **Compound** | **Compound ID** | **Description** | **Formula** | **Mass error** | **Isotope pattern** | **Score** | **Fold change (SA/Control)** | **p-value** |
| --- | --- | --- | --- | --- | --- | --- | --- | --- |
| 12.19_439.1226m/z | HMDB34157 | 2-Hydroxybenzaldehyde O-[xylosyl-(1->6)-glucoside] | C18H24O11 | 2.75 | 89.87 | 40.2 | 0.61 | 2.78E-02 |
| 11.16_379.2109m/z | HMDB41044 | 5,7-Megastigmadien-9-ol glucoside | C19H32O6 | 4.58 | 89.93 | 39 | 0.49 | 2.36E-03 |
| 11.45_208.0963m/z | HMDB40731 | Avenalumic acid | C11H10O3 | -2.95 | 94.05 | 41.7 | 0.31 | 3.92E-02 |
| 13.69_463.1831n | HMDB61137 | Dihydroisomorphine-6-glucuronide | C23H29NO9 | -2.05 | 90.70 | 46.1 | 5.85 | 6.14E-03 |
| 10.40_334.1491m/z | HMDB41024 | Hydroxytyrosol 1-O-glucoside | C14H20O8 | -2.45 | 84.19 | 41.9 | 0.29 | 1.33E-02 |
| 13.41_244.0842n | HMDB06005 | Indolylacryloylglycine | C13H12N2O3 | -2.36 | 92.74 | 50.7 | 3.43 | 1.05E-02 |
| 2.03_156.0763m/z | HMDB00177 | L-Histidine | C6H9N3O2 | -2.14 | 94.28 | 43.9 | 0.26 | 4.10E-02 |
| 13.24_595.3477m/z | HMDB04159 | L-Urobilin | C33H46N4O6 | -1.85 | 89.49 | 48.3 | 0.19 | 3.82E-02 |
| 6.92_198.0868m/z | HMDB32055 | N-Acetylhistidine | C8H11N3O3 | -2.37 | 94.57 | 42 | 0.22 | 3.51E-03 |
| 11.57_216.1589m/z | HMDB13279 | N-Nonanoylglycine | C11H21NO3 | -2.28 | 95.26 | 38.5 | 0.39 | 1.10E-02 |
| 13.73_381.1173m/z | HMDB29651 | Phlorisobutyrophenone 2-glucoside | C16H22O9 | 4.27 | 87.96 | 38.5 | 0.27 | 4.13E-02 |
| 2.10_255.1332m/z | HMDB29065 | Threoninyl-Leucine | C10H20N2O4 | 2.68 | 88.09 | 37.6 | 0.34 | 4.73E-02 |
| 11.23_404.0739n | HMDB60022 | Urolithin A-8-O-glucuronide | C19H16O10 | -1.63 | 92.92 | 48.3 | 0.16 | 3.26E-02 |

**Table S4** Information of potential biomarkers of SA prediction

| **Metabolite biomarker** | **AUC** | ***p*-value** | **Log2 FC** |
| --- | --- | --- | --- |
| Indolylacryloylglycine | 0.842 | 1.16E-02 | -4.76 |
| L-Histidine | 0.795 | 1.29E-03 | 1.83 |
| Avenalumic acid | 0.778 | 1.05E-02 | 3.49 |
| N-Acetylhistidine | 0.770 | 5.91E-03 | 6.84 |
| Dihydroisomorphine-6-glucuronide | 0.769 | 1.19E-02 | -5.16 |
| Hydroxytyrosol 1-O-glucoside | 0.762 | 1.33E-02 | 5.92 |
| 5,7-Megastigmadien-9-ol glucoside | 0.753 | 2.78E-02 | 4.68 |
| Phlorisobutyrophenone 2-glucoside | 0.752 | 3.03E-02 | 3.54 |
| L-Urobilin | 0.748 | 3.13E-02 | 9.87 |
| Threoninyl-Leucine | 0.726 | 4.98E-02 | 2.62 |
| N-Nonanoylglycine | 0.723 | 3.24E-02 | 2.89 |
